# Smart Microneedle Arrays Integrating Cell‐Free Therapy and Nanocatalysis to Treat Liver Fibrosis

**DOI:** 10.1002/advs.202309940

**Published:** 2024-06-14

**Authors:** Yanteng Xu, Yixin Zhang, Hao Tian, Qingguo Zhong, Ke Yi, Fenfang Li, Tiantian Xue, Haixia Wang, Yeh‐Hsing Lao, Yingying Xu, Yinxiong Li, Ling Long, Kai Li, Yu Tao, Mingqiang Li

**Affiliations:** ^1^ Laboratory of Biomaterials and Translational Medicine Center for Nanomedicine and Department of Ultrasound The Third Affiliated Hospital Sun Yat‐sen University Guangzhou 510630 China; ^2^ Department of Neurology The Third Affiliated Hospital Sun Yat‐sen University Guangzhou 510630 China; ^3^ Department of Pharmaceutical Sciences University at Buffalo The State University of New York Buffalo NY 14214 USA; ^4^ Center for Health Research Guangzhou Institutes of Biomedicine and Health Chinese Academy of Sciences Guangzhou 510530 China; ^5^ University of China Academy of Sciences Beijing 100049 China; ^6^ Guangdong Provincial Key Laboratory of Liver Disease Guangzhou 510630 China

**Keywords:** controlled release, liver fibrosis, microneedle array, nanozyme, stem cell secretome

## Abstract

Liver fibrosis is a chronic pathological condition lacking specific clinical treatments. Stem cells, with notable potential in regenerative medicine, offer promise in treating liver fibrosis. However, stem cell therapy is hindered by potential immunological rejection, carcinogenesis risk, efficacy variation, and high cost. Stem cell secretome‐based cell‐free therapy offers potential solutions to address these challenges, but it is limited by low delivery efficiency and rapid clearance. Herein, an innovative approach for in situ implantation of smart microneedle (MN) arrays enabling precisely controlled delivery of multiple therapeutic agents directly into fibrotic liver tissues is developed. By integrating cell‐free and platinum‐based nanocatalytic combination therapy, the MN arrays can deactivate hepatic stellate cells. Moreover, they promote excessive extracellular matrix degradation by more than 75%, approaching normal levels. Additionally, the smart MN arrays can provide hepatocyte protection while reducing inflammation levels by ≈70–90%. They can also exhibit remarkable capability in scavenging almost 100% of reactive oxygen species and alleviating hypoxia. Ultimately, this treatment strategy can effectively restrain fibrosis progression. The comprehensive in vitro and in vivo experiments, supplemented by proteome and transcriptome analyses, substantiate the effectiveness of the approach in treating liver fibrosis, holding immense promise for clinical applications.

## Graphical abstract

Advanced smart microneedle arrays, incorporating platinum‐based nanozymes with a photothermal effect, enable precise drug release. The responsively released core–shell nanoparticles encapsulating stem cell secretome deactivate hepatic stellate cells, rejuvenate hepatocytes, and inhibit inflammation. Additionally, the released nanozymes proficiently deplete reactive oxygen species and alleviate hypoxia. This sophisticated approach integrating cell‐free therapy and nanocatalysis demonstrates significant efficacy in treating liver fibrosis.

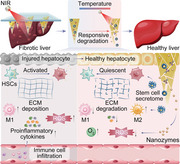

## 1 Introduction

Liver fibrosis, caused by factors such as viral infections, alcohol consumption, nonalcoholic steatosis, drug abuse, and autoimmunity, represents a significant global health issue.^[^ ^1^ ^]^ In its early stages, damage to hepatic parenchymal cells prompts the release of profibrotic cytokines and growth factors by hepatocytes, Kupffer cells, and sinusoidal endothelial cells. This leads to the activation of hepatic stellate cells (HSCs), which proliferate persistently and secrete large amounts of extracellular matrix (ECM)‐associated proteins, causing excessive ECM accumulation and fibrogenesis.^[^ ^2^ ^]^ Concurrently, the fibrosis‐induced hypoxic environment inhibits the regeneration of damaged hepatocytes, aggravating hepatocellular injury.^[^ ^3^ ^]^ In addition, the ongoing production of damage‐associated molecular patterns and chemokines not only induces a pro‐inflammatory M1 phenotype in Kupffer cells but also recruits neutrophils and monocytes to the liver, where the latter can differentiate into macrophages and undergo further M1 polarization. The M1 macrophages and infiltrated neutrophils secrete proinflammatory cytokines and chemokines, further exacerbating liver fibrosis and inducing hepatic damage.^[^ ^4^ ^]^ Without prompt intervention, liver fibrosis may advance to cirrhosis or hepatocellular carcinoma, leading to end‐stage liver disease. Despite its severity, there are currently no direct therapy approaches available for the treatment of liver fibrosis.^[^ ^4^, ^5^ ^]^

Stem cell‐based tissue engineering and regenerative medicine have garnered increasing attention due to their potential in promoting tissue and organ repair.^[^ ^6^ ^]^ However, the clinical applications of stem cell therapy are hindered by challenges such as immunological rejection, potential carcinogenesis, variable treatment efficacy, and exorbitant costs.^[^ ^7^ ^]^ A key mechanism of stem cells’ reparative effects is their paracrine action.^[^ ^6^, ^8^ ^]^ The stem cell secretome, comprising a range of cytokines and extracellular vesicles (such as microvesicles and exosomes), exhibits multifaceted biological activities, particularly in tissue repair and inflammation reduction.^[^ ^9^ ^]^ Additionally, stem cell secretome‐based cell‐free therapy not only overcomes the limitations associated with stem cell therapy but also shows promising therapeutic efficacy in treating hepatic disorders.^[^ ^10^ ^]^ Nanozymes, as the burgeoning therapeutic agents, often exhibit remarkable antioxidant and anti‐inflammatory capabilities, characterized by their high catalytic activity, exceptional stability, cost‐effectiveness in preparation, and resistance to long‐term storage. These properties effectively overcome the complexities associated with natural enzyme preparation and purification. To date, nanozymes have found extensive applications in biological detection and anti‐inflammatory therapy.^[^ ^11^ ^]^ A recent report demonstrated the efficacy of a heart‐targeting Fe‐Cur@TA nanoenzyme, which exhibited remarkable capabilities in scavenging free radicals and reducing inflammation by inhibiting immune cell infiltration.^[^ ^11b^ ^]^ Additionally, this nanoenzyme promoted macrophage polarization toward an M2 phenotype while suppressing inflammatory cytokine secretion and impeding the circulation of inflammatory free radicals. In the management of liver‐related diseases, CuNZ nanoenzymes were confirmed to effectively treat liver failure.^[^ ^12^ ^]^ Both in vitro and in vivo experiments demonstrated that this nanozyme could mitigate reactive oxygen species (ROS) levels during the early stages of liver failure while concurrently reducing the accumulation of pro‐inflammatory cytokines, thereby preventing the progression of hepatocyte necrosis. These antioxidant nanoenzymes provide innovative perspectives and potential strategies for the treatment of diseases associated with inflammation. Besides, previous reports have demonstrated that platinum‐based nanoenzymes (PtNZs) exhibit extensive and efficient catalytic capabilities in reducing inflammation primarily through scavenging ROS.^[^ ^13^ ^]^ They possess the ability to expedite the decomposition of H_2_O_2_ into O_2_ and H_2_O.^[^ ^13a^ ^]^ Furthermore, PtNZs can mimic the function of superoxide dismutase (SOD) in neutralizing •O_2_^−^.^[^ ^13b^ ^]^ Thus, the application of PtNZs holds significant therapeutic implications in the therapy of liver fibrosis. Despite the great potential of stem cell secretome and PtNZs, the current delivery methods, such as local injection at the injury site and systemic administration, face challenges in terms of rapid clearance rate and low efficiency.^[^ ^14^ ^]^ Therefore, it is imperative to develop more effective delivery vectors to enhance the duration and bioavailability of the therapeutic agents, thereby improving therapeutic outcomes.

The microneedle (MN) array is regarded as a promising drug delivery platform due to its precise, highly efficient, and painless characteristics.^[^ ^15^ ^]^ The constituents of MNs greatly determine their degradation rate and drug release behaviors in vivo.^[^ ^16^ ^]^ The soy protein isolate (SPI), a widely sourced natural protein extracted from soybeans, exhibits excellent biocompatibility, biodegradability, and processability, rendering it extensively applied as hydrogels, films, or emulsions in the field of biotechnology and biomaterials.^[^ ^12^, ^17^ ^]^ Its plant‐based nature ensures gradual degradation within animal tissues, making it an attractive candidate for incorporation in MN arrays to achieve extended drug retention.

**Scheme 1:**
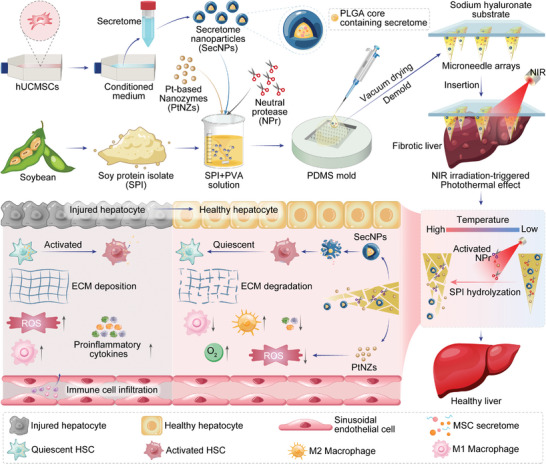
Schematic illustration of fabricating and implanting NIR‐responsive MN arrays containing hUCMSC‐derived secretome‐encapsulated core–shell nanoparticles and versatile nanozymes to alleviate liver fibrosis. The conditioned medium of hUCMSCs, devoid of serum, was collected and subjected to purification to obtain the secretome, which was then encapsulated in the PLGA core of SecNPs using a double emulsification technique. The SecNPs and PtNZs were integrated into smart MN arrays consisting of SPI, PVA, and NPr. The MN patches were implanted into the fibrotic liver and underwent responsive degradation through the hydrolysis of NPr, which was activated by the photothermal effect of PtNZs upon NIR irradiation. The released SecNPs could repair injured hepatocytes, promote hepatocyte proliferation, suppress M1 polarization while enhancing M2 polarization, attenuate the secretion or infiltration of proinflammatory cytokines, inhibit HSC activation, induce the quiescence of activated HSCs, prevent excessive ECM deposition, and facilitate ECM degradation. The released PtNZs could transform or deplete ROS and generate O_2_.

We hereby present a cutting‐edge approach for the in situ implantation of smart MN arrays that seamlessly integrate cell‐free therapy and platinum‐based nanocatalytic therapy. The smart MN patch possesses the capability to perforate the liver capsule and deliver multiple therapeutic agents directly into the fibrotic liver tissue in a controlled and responsive manner, for liver fibrosis treatment (**Scheme** **1**). The MNs were fabricated by blending heat‐induced structure‐unfolded SPI with polyvinyl alcohol (PVA), incorporating stem cell secretome‐encapsulated core–shell nanoparticles (SecNPs), versatile PtNZs, and neutral protease (NPr). Following the in situ implantation of our crafted MN patches, the PtNZs within MNs exerted their exceptional efficacy in converting near‐infrared (NIR) irradiation into thermal energy. This photothermal effect led to an elevation in local temperature at the implantation site, enabling it to reach the optimal catalysis temperature of NPr, and thereby activating this protease. The activated NPr effectively catalyzed SPI hydrolysis, further inducing the responsive degradation of MNs and controlled releases of PtNZ and SecNPs, ultimately resulting in a long‐lasting and precise therapeutic effect on the fibrotic liver. The released SecNPs were internalized by the HSCs, hepatocytes, and macrophages within the liver, subsequently deactivating HSCs, inhibiting excessive ECM deposition, promoting ECM degradation, safeguarding hepatocytes against damage, facilitating hepatocyte proliferation, and alleviating inflammatory responses. Meanwhile, the released PtNZs could efficiently scavenge excess ROS and mitigate the hypoxic microenvironment caused by the fibrosis progression, thereby conducive to the SecNPs‐mediated liver tissue repair and regeneration. Hence, our responsive MN arrays provide a promising strategy for achieving notable therapeutic efficacy in the treatment of fibrotic livers through NIR‐responsive and controlled release of SecNPs and PtNZs, demonstrating remarkable potential for clinical application.

## 2 Results

### 2.1 Fabrication and Characterization of SecNPs

**Figure 1:**
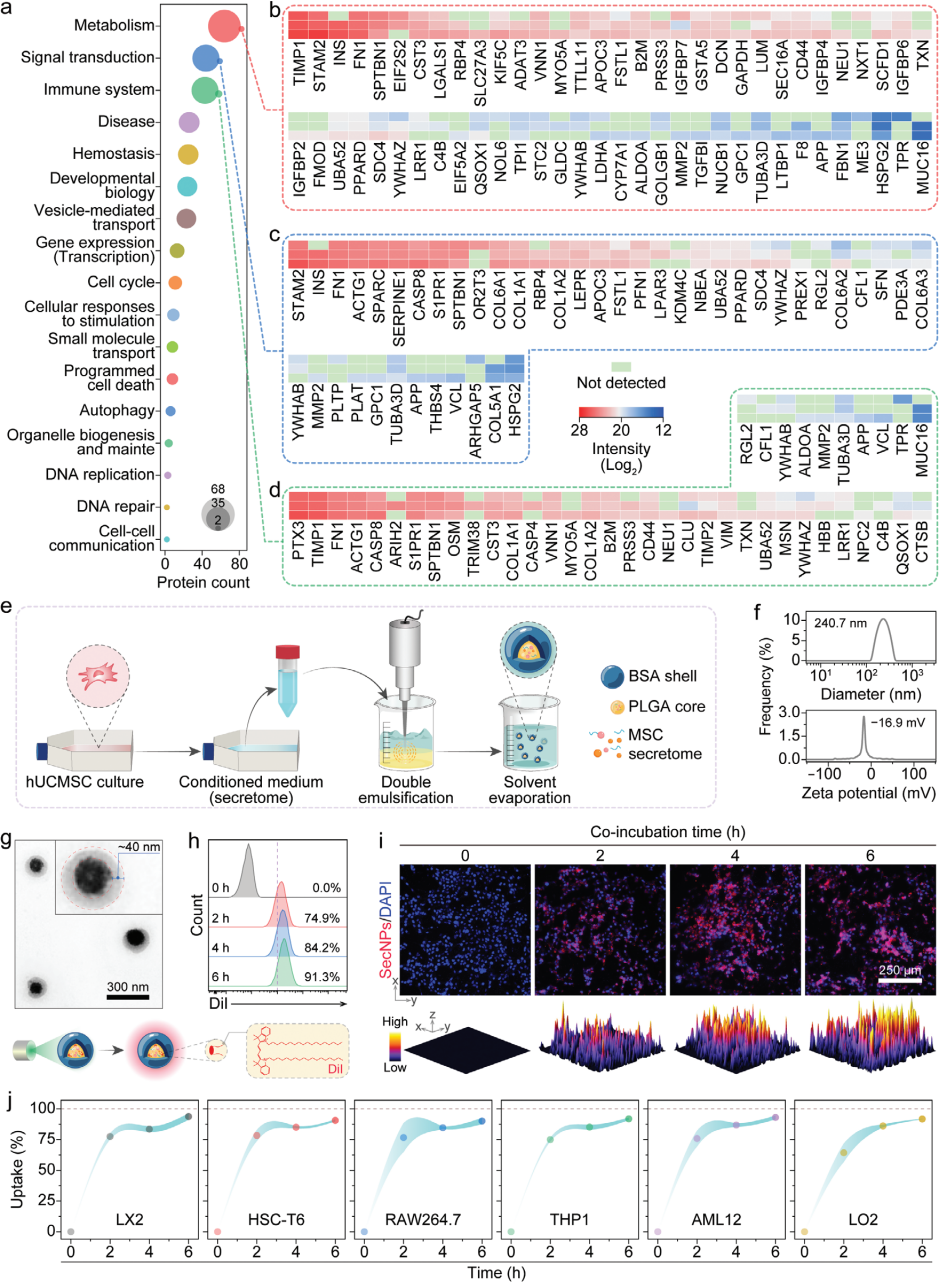
Fabrication, characterization, and property of SecNPs. a) Reactome enrichment based on label‐free proteome analysis of secretome derived from hUCMSCs. b–d) Heatmap displaying the proteins and their abundances in hUCMSC secretome involved in metabolism (b), signal transduction (c), and immune system (d). e) Schematic illustration of fabricating SecNPs. f) Hydrodynamic diameter distribution and zeta potential of SecNPs. g) Representative TEM image indicating remarkable core–shell microstructure of SecNPs. h) Representative flow cytometry histograms depicting time‐dependent uptake of SecNPs by LX2 cells. i) Representative fluorescence images and corresponding quantified 3D surface plots indicating the internalization of DiI‐labeled SecNPs by HSC‐T6 cells over various co‐incubation periods. j) Summarized time‐dependent uptake curves of SecNPs by various cells. Data are presented as mean ± standard deviation (SD), *n* = 3.

The serum‐free conditioned medium culturing human umbilical cord mesenchymal stem cells (hUCMSCs) was collected, followed by centrifugation, filtration, and lyophilization to obtain the purified secretome (Figure S1, Supporting Information). Label‐free proteome sequencing and analysis of the hUCMSC secretome identified 186 discernible proteins associated with metabolism, signal transduction, immune system regulation, disease pathogenesis, hemostasis mechanisms, developmental biology processes, vesicle‐mediated transport phenomena, and other related areas (Figures S2–S4, Supporting Information). The top three Reactome pathways, namely metabolism, signal transduction, and the immune system displayed remarkable associations with the biological regulation of improving liver function and suppressing inflammation (**Figure** **1**a–d). Revealing the protein constituents and latent functions provided us with the impetus for harnessing the hUCMSC secretome to ameliorate liver fibrosis.

To maximize the preservation of functional proteins in the hUCMSC secretome and facilitate their efficient cellular uptake for optimal regulatory effects, we encapsulated the hUCMSC secretome within the core of SecNPs (Figure 1e), using double emulsification and solvent evaporation techniques (Figure S1, Supporting Information). The obtained SecNPs exhibited a secretome loading content of ≈4.74 µg mg^–1^, with a loading efficiency of ≈97.22%. According to the results obtained separately from dynamic light scattering (DLS) and electrophoretic light scattering (ELS) assays (Figure 1f), the hydrodynamic diameter of SecNPs exhibited a distribution centered ≈240.7 nm, while the zeta potential of SecNPs demonstrated an average value of ≈−16.9 mV. As determined by transmission electron microscopy (TEM), SecNPs exhibited a characteristic double‐layered core–shell spherical morphology (Figure 1g), with a particle size of ≈250 nm and a shell thickness of ≈40 nm.

After confirming the physical properties of SecNPs, we further investigated their cellular uptake by employing a fluorescence labeling technique through loading 1,1′‐di‐n‐octadecyl‐3,3,3′,3′‐tetramethylindocarbocyanine perchlorate (DiI) in the poly (lactic‐co‐glycolic acid) (PLGA) core. As shown in Figure 1h (LX2 cells) and 1i (HSC‐T6 cells), the DiI‐labeled SecNPs could be time‐dependently internalized by HSCs. The flow cytometry analyses (Figure 1h,j; Figure S5a, Supporting Information) and microscopic images (Figure 1i) clearly revealed that nearly all the LX2 and HSC‐T6 cells exhibited DiI‐positive fluorescence after the co‐incubation for 6 h, indicating an uptake efficiency close to 100% (91.3% for LX2 cells, Figure 1h; 92.6% for HSC‐T6 cells, Figure S5a, Supporting Information). Comparable uptake efficiencies of SecNPs were also observed in other cell types (Figure 1j) including macrophages (92.3% for RAW264.7 cells, Figure S6a, Supporting Information); 93.1% for THP1 cells, Figure S6b, Supporting Information) and hepatocytes (93.8% for AML12 cells, Figure S7a,b, Supporting Information; 92.1% for LO2 cells, Figure S7c,d, Supporting Information), which foreboded the efficient internalization of our engineered SecNPs within liver tissues.

### 2.2 In Vitro Antifibrotic Effect of SecNPs

**Figure 2:**
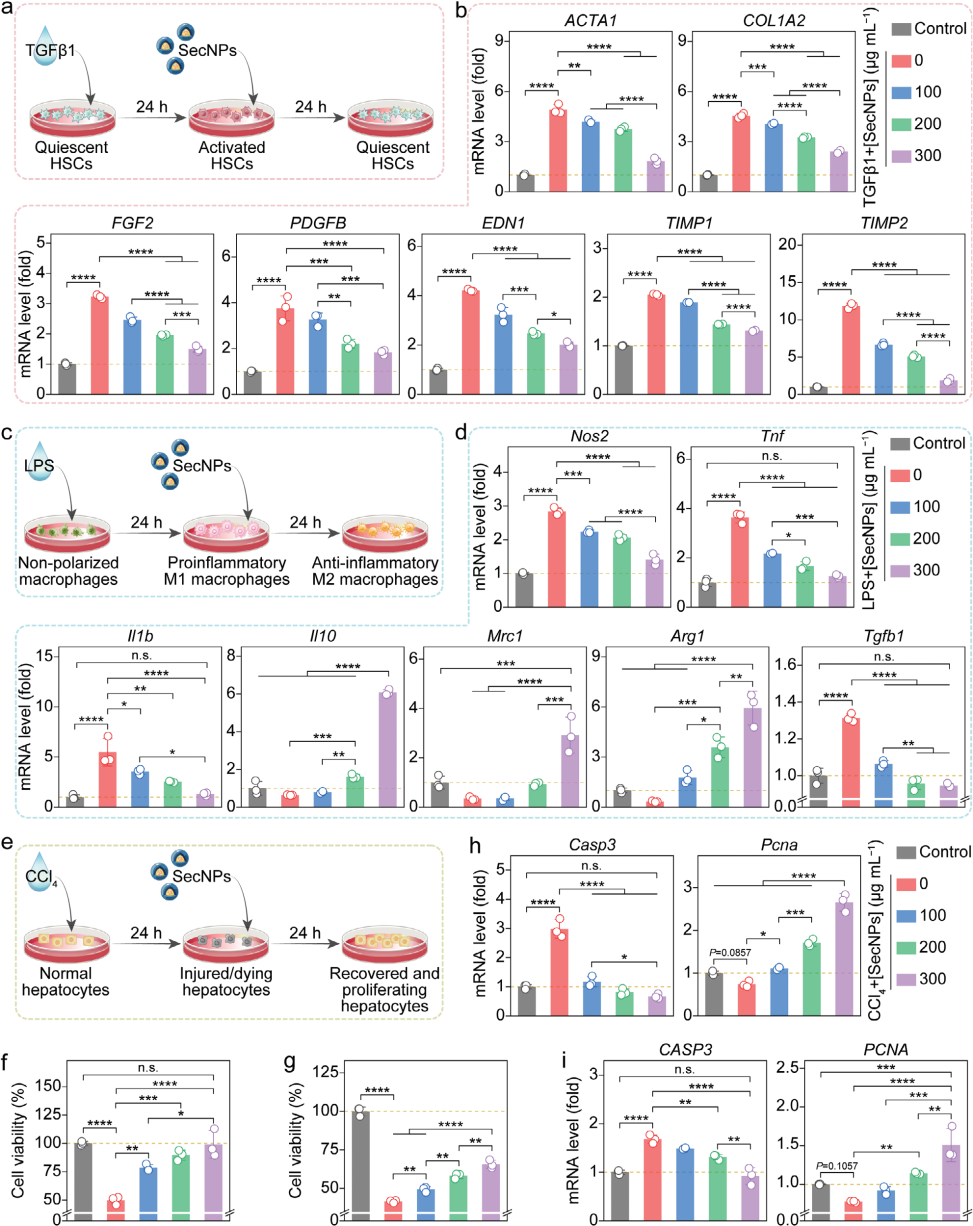
In vitro inhibition of liver fibrosis‐related pathological process by SecNPs. a) Schematic illustration of employing SecNPs to treat TGFβ1‐activated hepatic stellate cells (HSCs). b) Relative intracellular levels of mRNA implicated in ECM deposition (*ACTA1* and *COL1A1*), HSC activation (*FGF2*, *PDGFB*, and *END1*), and ECM degradation (*TIMP1* and *TIMP2*) evaluated using LX2 cells. c) Schematic representation of using SecNPs to treat LPS‐activated M1 macrophages. d) Relative intracellular mRNA levels of proinflammatory markers *(Nos2*, *Tnf*, and *Il1b*), anti‐inflammatory markers (*Il10*, *Mrc1*, and *Arg1*), or profibrotic marker (*Tgfb1*) assessed using RAW264.7 cells following the indicated treatments. e) Schematic diagram of using SecNPs to treat CCl_4_‐induced injured hepatocytes. f,g) Cell viabilities of AML12 (f) and LO2 (g) cells undergoing the sequential co‐incubations with CCl_4_ and SecNPs. h, i) Relative intracellular levels of mRNA associated with apoptosis (h, *Casp3*; i, *CASP3*) or proliferation (h, *Pcna*; i, *PCNA*) determined employing AML12 (h) and LO2 (i) cells subjected to the indicated treatments. Data are presented as mean ± SD (*n* = 3). Statistical significances were assessed using one‐way analysis of variance (ANOVA) followed by Tukey's multiple comparisons post hoc test. ^*^ *p* < 0.05; ^**^ *p* < 0.01; ^***^ *p* < 0.001; ^****^ *p* < 0.0001; and n.s., not significant.

During the initial stages of liver fibrosis, HSCs receive molecular signals from neighboring cells that can secrete various growth factors, thereby activating HSCs and promoting profibrotic factor secretion.^[^ ^1b^ ^]^ Subsequently, the activated HSCs proliferate and secrete substantial quantities of ECM proteins, leading to ECM accumulation and fibrous scar formation.^[^ ^18^ ^]^ To investigate the anti‐fibrotic effect of SecNPs, LX2 or HSC‐T6 cells were initially activated by the addition of a typical fibroblast growth factor, TGFβ1, followed by co‐incubation with SecNPs (**Figure** **2**a). Then, the expression levels of fibrosis‐associated genes were quantified using real‐time quantitative polymerase chain reaction (RT‐qPCR) assays. After internalization by HSCs (Figure 1h,j; Figure S5a, Supporting Information), SecNPs effectively normalized the aberrant gene expressions induced by TGFβ1 stimulation in LX2 (Figure 2b) and HSC‐T6 cells (Figure S5b, Supporting Information). These genes included smooth muscle actin alpha 1 (*ACTA1*, for LX2 cells), smooth muscle actin alpha 2 (*Acta2*, for HSC‐T6 cells), collagen type 1 alpha 2 (*COL1A2*, for LX2 cells), fibroblast growth factor 2 (*FGF2*, for LX2 cells; *Fgf2*, for HSC‐T6 cells), platelet‐derived growth factor subunit A (*Pdgfa*, for HSC‐T6 cells), platelet‐derived growth factor subunit B (*PDGFB*, for LX2 cells; *Pdgfb*, for HSC‐T6 cells), *Tgfb1* (for HSC‐T6 cells), endothelin 1 (*EDN1*, for LX2 cells), tissue inhibitor of metalloproteinase 1 (*TIMP1*, for LX2 cells; *Timp1*, for HSC‐T6 cells), tissue inhibitor of metalloproteinase 2 (*TIMP2*, for LX2 cells; *Timp2*, for HSC‐T6 cells), and matrix metalloproteinase 13 (*Mmp13*, for HSC‐T6 cells). These findings implied that SecNPs could effectively suppress profibrotic signaling, leading to the deactivation of HSCs.

### 2.3 In Vitro Anti‐Inflammatory Effect of SecNPs

Inflammation plays a critical role in the pathogenesis and progression of liver fibrosis, and prolonged inflammatory stimuli can transform liver tissue repair into fibrotic development.^[^ ^4^, ^19^ ^]^ Immune cells, particularly Kupffer cells, and recruited macrophages, are key regulators of liver inflammation.^[^ ^20^ ^]^ Proinflammatory macrophages secrete various cytokines that activate HSCs and damage liver parenchyma.^[^ ^21^ ^]^ The rapid internalization of SecNPs by RAW264.7 and THP1 cells has been demonstrated (Figure 1j; Figure S6a,b, Supporting Information). To investigate the potential of SecNPs in modulating macrophage polarization from M1 (proinflammatory phenotype) to M2 (anti‐inflammatory phenotype), we quantified the intracellular mRNA levels of representative genes in SecNPs‐treated lipopolysaccharide (LPS)‐stimulated RAW 264.7 (Figure 2d) and THP1 (Figure S6c, Supporting Information) cells. The results demonstrated a significant decrease in the expression levels of proinflammatory cytokines, including nitric oxide synthase 2 (*Nos2*, for RAW264.7 cells; *NOS2*, for THP1 cells), tumor necrosis factor (*Tnf*, for RAW264.7 cells), and interleukin 1 beta (*Il1b*, for RAW264.7 cells) in macrophages treated successively with LPS and SecNPs, compared to those treated with LPS alone (Figure 2d; Figure S6c, Supporting Information). Reassuringly, the expression levels of *NOS2*, *Tnf*, and *Il1b* in macrophages treated with LPS followed by SecNPs were restored to their basal levels without any exogenous stimulus.

Meanwhile, the levels of anti‐inflammatory cytokines, such as interleukin 10 (*Il10*, for RAW264.7 cells; *IL10*, for THP1 cells), mannose receptor C 1 (*Mrc1*, for RAW264.7 cells), and arginase 1 (*Arg1*, for RAW264.7 cells), significantly increased in macrophages following the incubation with SecNPs (Figure 2d; Figure S6c, Supporting Information). Additionally, SecNPs resulted in the downregulation of transforming growth factor beta 1 (*Tgfβ1*, for RAW264.7 cells; *TGFB1*, for THP1 cells), a typical cytokine known to activate HSCs and promotes their myofibroblast transition, which was significantly upregulated upon the stimulation with LPS (Figure 2d; Figure S6c, Supporting Information). Moreover, the expression level of matrix metalloproteinase 9 (*MMP9*, for THP1 cells), a crucial cytokine responsible for collagen hydrolysis and ECM degradation, obviously increased in SecNP‐treated macrophages compared to M1‐polarized macrophages (Figure S6c, Supporting Information). Hence, SecNPs exhibited notable potential in ameliorating the concurrent inflammation associated with liver fibrosis.

### 2.4 In Vitro Hepatoprotective Effect of SecNPs

The progression of liver fibrosis is closely associated with hepatocellular injury. The stem cell secretome contains various bioactive factors that can effectively mitigate cellular damage and facilitate the repair and proliferation of hepatocytes.^[^ ^22^ ^]^ To investigate the hepatoprotective effect, AML12 or LO2 cells were exposed to CCl_4_ to simulate in vivo liver injury, followed by co‐incubation with varying concentrations of SecNPs (Figure 2e). Cell viabilities were determined using CCK8 assays, and the results are shown in Figure 2f (AML12 cells) and Figure 2g (LO2 cells). CCl_4_ treatment significantly reduced hepatocyte viability and proliferation, but this effect was notably reversed by SecNPs. Besides, SecNPs exhibited a concentration‐dependent protective effect on the injured hepatocytes. Remarkably, following the co‐incubation with SecNPs at a concentration of 300 µg mL^−1^ for 24 h, the cell viability of CCl_4_‐treated AML12 cells was ≈100% restored to the levels of undamaged cells (Figure 2f).

The restorative and proliferative capabilities of SecNPs on CCl_4_‐damaged hepatocytes were further validated through molecular mechanism analysis using RT‐qPCR techniques (Figure 2h,i). It was evident that the intracellular mRNA level of an apoptosis‐promoting gene Caspase 3 (*Casp3* for AML12 cells, Figure 2h; *CASP3* for LO2 cells, Figure 2i) was significantly reduced upon the rescue by SecNPs, accompanied by a prominent increase in the intracellular mRNA level of proliferating cell nuclear antigen (*Pcna* for AML12 cells, Figure 2h; *PCNA* for LO2 cells, Figure 2i). Particularly, the mRNA abundance of *Pcna* or *PCNA* in the hepatocytes undergoing the co‐incubation with SecNPs at a concentration of 300 µg mL^–1^ was 1.5–3 times higher than that in normal hepatocytes, indicating a notable enhancement effect of SecNPs on hepatocyte proliferation.

Given the responsive release of SecNPs at a temperature of 45 °C, induced by NIR irradiation, an investigation was conducted to assess their functions at this elevated temperature. The hepatocyte viability restoration and proliferation facilitation functions of SecNPs remained considerable (Figure S8, Supporting Information), even after prolonged incubation at 45 °C for 30 min. Notably, the heated SecNPs effectively restored AML12 cell viability to levels comparable with those of normal AML12 cells (Figure S8a, Supporting Information). Consequently, all these results demonstrated the robust hepatoprotective capabilities of SecNPs and their potential to facilitate hepatocyte proliferation within the microenvironment of liver fibrosis.

### 2.5 Synthesis and Characterization of PtNZs

**Figure 3:**
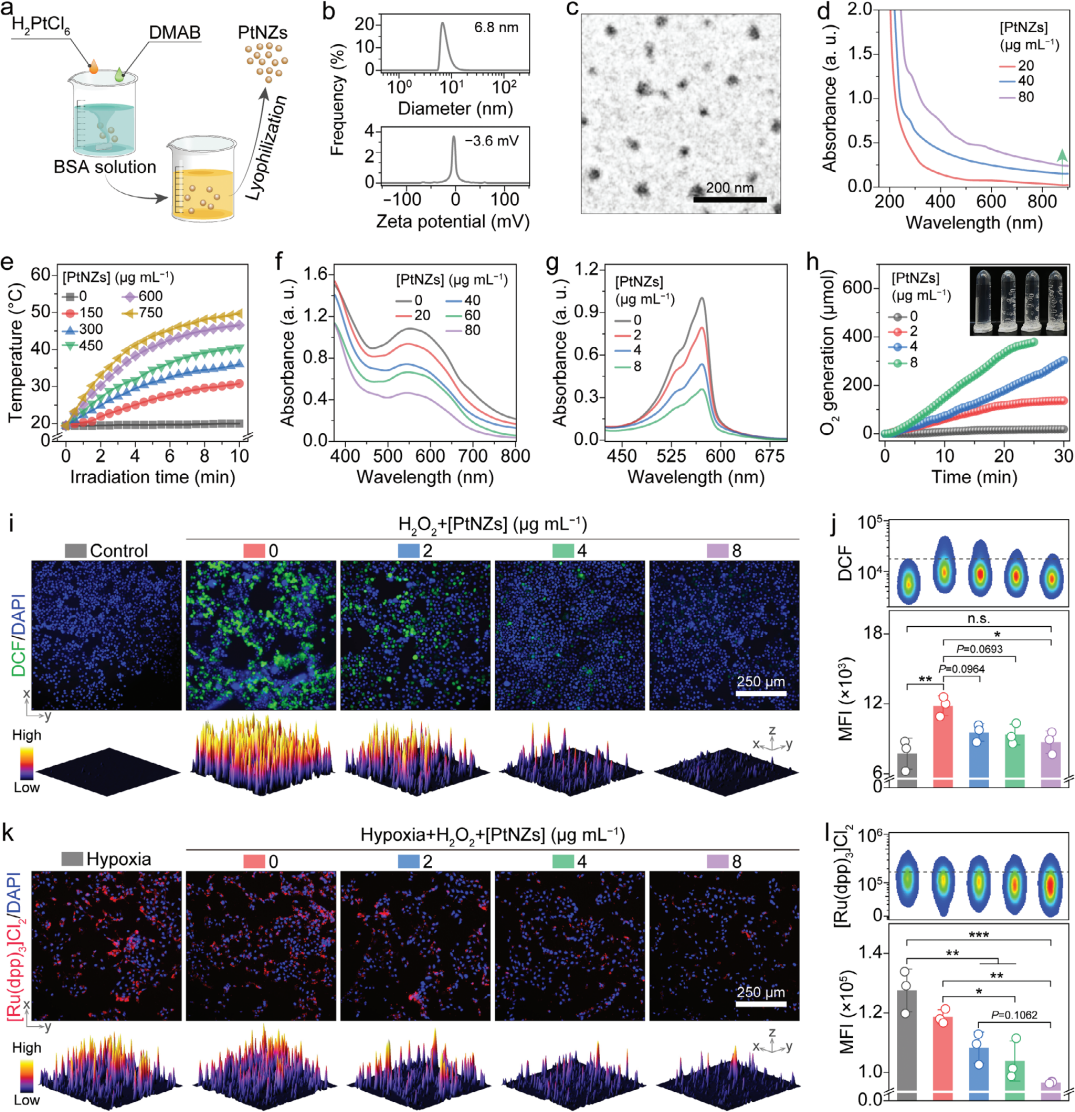
Synthesis, characterization, property, and in vitro hepatoprotective effect of PtNZs. a) Schematic illustration of the PtNZ synthetic process. b) Hydrodynamic diameter distribution and zeta potential of PtNZs. c) Representative TEM image of PtNZs. d) UV–vis absorption spectrum of PtNZs. e) Photothermal effect of PtNZs at various concentrations under NIR irradition. f) SOD‐mimicking activity of PtNZs manifested by the depletion of •O_2_^–^. g,h) CAT‐like activity of PtNZs evidenced by H_2_O_2_ conversion (g) and O_2_ generation (h). i,j) Intracellular ROS‐scavenging activity of PtNZs evaluated using representative DCF^+^ fluorescence images (i) accompanied by quantified 3D surface plots and representative DCF^+^ flow cytometry pseudo‐color plots with corresponding quantifications (j) in AML12 cells following the indicated treatments. k,l) Intracellular capability of PtNZs for catalyzing H_2_O_2_ into O_2_ estimated by representative [Ru(dpp)_3_]Cl_2_^+^ (reflecting the hypoxia level) fluorescence images (k) with quantified 3D surface plots and representative [Ru(dpp)_3_]Cl_2_^+^ flow cytometry pseudo‐color plots with corresponding quantifications (l) in LX2 cells undergoing the indicated treatments. Data are shown as mean ± SD (*n* = 3). Statistical significances were assessed by one‐way ANOVA with Tukey's multiple comparisons post hoc test. ^*^ *p* < 0.05; ^**^ *p* < 0.01; ^***^ *p* < 0.001; and n.s., not significant.

The PtNZs were synthesized through the reduction of H_2_PtCl_6_ in the presence of bovine serum albumin (BSA), followed by lyophilization (**Figure** **3**a). Detected by DLS and ELS techniques, the PtNZs exhibited a particle size of ≈6.8 nm and a zeta potential of ≈−3.6 mV, respectively (Figure 3b). The TEM image depicted a spherical morphology for the PtNZs (Figure 3c). The incorporation of PtNZs into MNs enabled the utilization of their photothermal conversion property to elevate the local temperature at the liver tissue implantation site, thereby promoting MN degradation through enhanced NPr enzyme activity.^[^ ^23^ ^]^ The UV–vis spectrophotometry analysis revealed an enhanced absorption of PtNZs at 808 nm with increasing PtNZ concentration (Figure 3d). Additionally, the photothermal effects of PtNZs were evident as the temperature of PtNZ solutions rose upon NIR irradiation (808 nm, 1 W cm^–2^) for 10 min, positively correlating with PtNZ concentration (Figure 3e).

The pathological context of liver fibrosis involves excessive ROS production, prompting the need for ROS elimination in fibrosis treatment.^[^ ^24^ ^]^ Pt‐based nanoparticles commonly exhibit nanozyme activity in addition to their photothermal conversion properties.^[^ ^25^ ^]^ Herein, the SOD‐like property of PtNZs was evaluated by monitoring •O_2_^−^ depletion, using a blue chloride (NBT) method at different PtNZ concentrations (Figure 3f). The reduction of NBT was induced by •O_2_^−^, resulting in an absorption peak at ≈560 nm. By scavenging •O_2_^−^, the SOD‐mimicking PtNPs effectively inhibited the reduction of NBT. The observed decrease in the absorption peak confirmed the effective SOD‐like activity of PtNPs (Figure 3f). Besides •O_2_^−^, H_2_O_2_ is another important ROS component. The depletion of H_2_O_2_ was confirmed using an Amplex red assay in the presence of different concentrations of PtNZs (Figure 3g) or at varying time intervals (Figure S9, Supporting Information). The results reflected that the effectiveness of PtNZs in scavenging H_2_O_2_ exhibited a positive correlation with both PtNZ concentration and duration. At a concentration of 2 µg mL^–1^, the H_2_O_2_ elimination rate was measured at 21%, while an increase in PtNZ concentration to 8 µg mL^−1^ resulted in a clearance rate of 66%. The PtNZ‐ concentration‐dependent O_2_ generation provided further evidence for the catalytic activity resembling that of catalase (CAT) exhibited by PtNZs (Figure 3h). These results offered compelling evidence for the exceptional SOD‐ and CAT‐like activities of PtNZs in vitro.

### 2.6 In Vitro Multienzyme‐Mimicking Activity of PtNZs

During liver fibrosis development, the excessive production of ROS caused by oxidative stress triggers hepatocyte apoptosis or necrosis, thereby accelerating disease progression.^[^ ^19^ ^]^ Therefore, it is crucial to mitigate ROS levels in the liver.^[^ ^26^ ^]^ Herein, AML12 or LO2 cells were initially exposed to H_2_O_2_, followed by co‐incubation with varying concentrations of PtNZs. Then, the intracellular ROS levels were assessed using a cell‐permeable fluorescence probe, namely 2′,7′‐dichlorodihydrofluorescein‐diacetate (DCFH‐DA), of which the DA group could be hydrolyzed by the intracellular esterase, leading to the oxidation of DCFH and the generation of green fluorescence‐producing DCF. After the treatment with H_2_O_2_, the hepatocytes exhibited a reduction in numbers and displayed an aberrant morphology (Figure 3i,k; Figures S10a, Supporting Information). Importantly, a clear trend of decreasing intracellular ROS levels was observed with increasing concentrations of PtNZs. The ROS generated in either AML12 (Figure 3i) or LO2 (Figure S10a, Supporting Information) cells upon treatment with H_2_O_2_ was effectively eliminated after a 12‐h co‐incubation with 8 µg mL^−1^ PtNZs. The potency of PtNZs in ROS elimination was further validated through flow cytometry analyses (Figure 3j; Figure S10b, Supporting Information). Subsequently, the mean fluorescence intensity (MFI) of DCF in the AML12 (Figure 3j) or LO2 (Figure S10b, Supporting Information) cells successively treated with PtNZs (8 µg mL^−1^) and H_2_O_2_ returned to the baseline levels of untreated cells.

The HSC activation can be further facilitated by hypoxia, which is a prevalent characteristic in the progression of liver fibrosis.^[^ ^3^, ^27^ ^]^ In vitro experiments demonstrated the catalytic ability of PtNZs in decomposing H_2_O_2_ to generate O_2_ (Figure 3h). Then, the intracellular generation of O_2_ by PtNZs in LX2 or HSC‐T6 cells was further confirmed using tris (4,7‐diphenyl‐1,10‐phenanthroline) ruthenium (II) dichloride complex ([Ru(dpp)_3_]Cl_2_), an oxygen‐specific probe that can be quenched by O_2_. After pre‐culturing in a hypoxic environment, the fluorescence intensity of [Ru(dpp)_3_]Cl_2_ in LX2 (Figure 3k) or HSC‐T6 (Figure S11a, Supporting Information) cells treated with H_2_O_2_ alone slightly decreased, suggesting a minor O_2_ production due to the self‐decomposition of internalized H_2_O_2_. Remarkably, for the LX2 (Figure 3k) or HSC‐T6 (Figure S11a, Supporting Information) cells co‐incubation with H_2_O_2_ and PtNZs (8 µg mL^−1^), the red fluorescence significantly declined, signifying effective intracellular H_2_O_2_ decomposition into O_2_ by PtNZs. The flow cytometry analyses also displayed the same variation trends as observed in the fluorescence of intracellular [Ru(dpp)_3_]Cl_2_ (Figure 3l; Figure S11b, Supporting Information). These findings underscore the significant therapeutic potential of PtNZs for liver fibrosis treatment.

### 2.7 Construction and Characterization of MN Arrays

**Figure 4:**
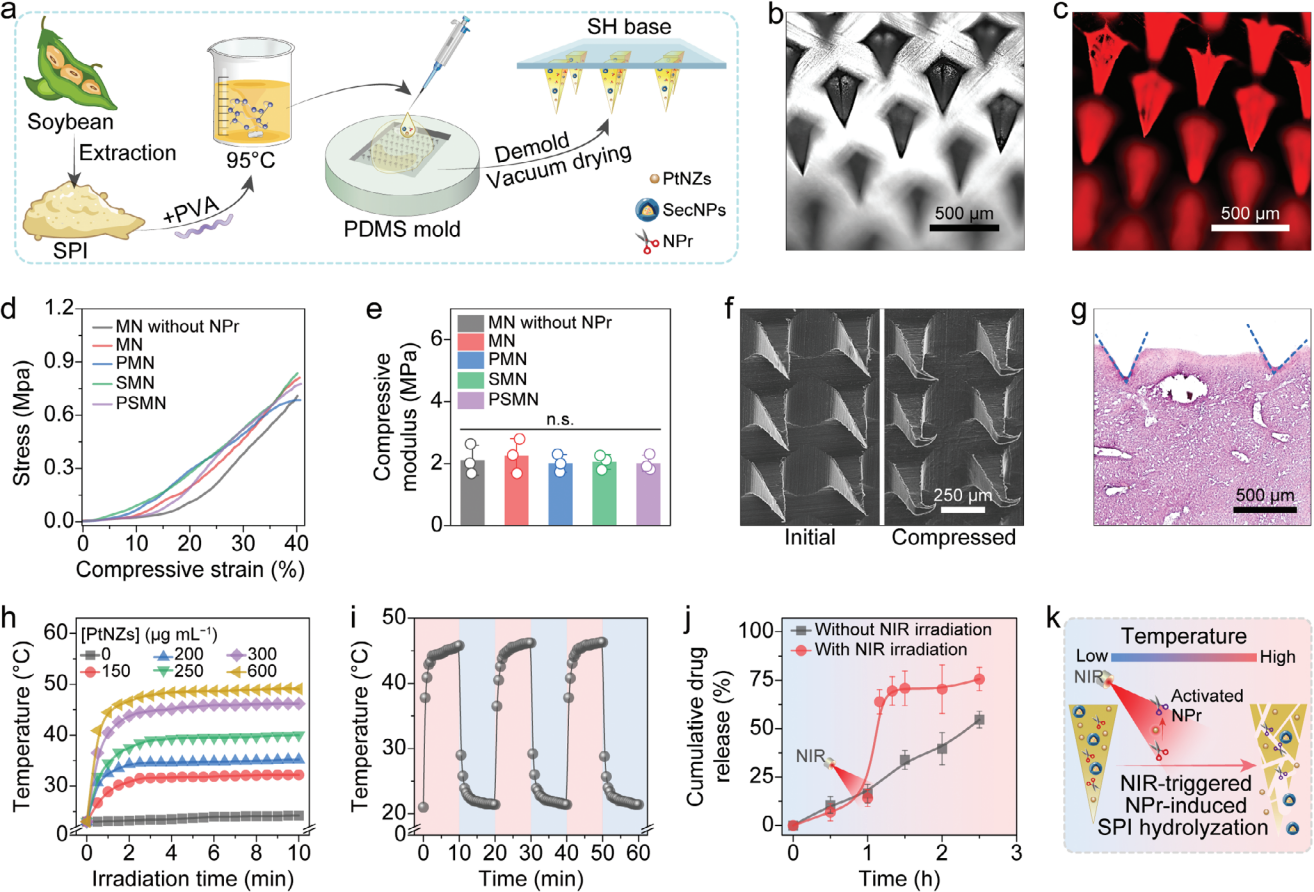
Fabrication and characterization of MN arrays. a) Schematic diagram of fabricating MN arrays with SH substrates and SPI‐PVA needle tips loaded with SecNPs, PtNZs, or NPr. b,c) Representative images of MNs in bright (b) and fluorescent (c) fields. d,e) Mechanical strength of various MNs evaluated by compressive stress‐strain curves (d) and corresponding quantified compressive moduli (e). f) Representative SEM images of MNs prior to and following the compression test. g) Representative histopathological image of H&E‐stained liver tissue post MN insertion. h) Photothermal effect of PMN arrays containing different concentrations of PtNZs under NIR irradition. i) Temperature variations of PMN arrays over three NIR irradiation on/off cycles. j) Cumulative release profiles of PSMN arrays with or without the irradiation of NIR. k) Schematic illustration of NIR‐triggered NPr activation and SPI hydrolyzation by the activated NPr. Data are presented as mean ± SD (*n* = 3). Statistical significances were assessed by one‐way ANOVA with Tukey's multiple comparisons post hoc test. n.s., not significant.

SPI, a plant protein derived from soybeans, is noted for its exceptional biocompatibility, biodegradability, and processability, making it an ideal candidate for responsively degradable material in drug delivery and tissue engineering.^[^ ^17^ ^]^ This protein can be hydrolyzed by NPr, which is effectively activated at appropriately high temperature through NIR irradiation. Surprisingly, we observed a significant enhancement in hepatocyte proliferation upon the treatment with SPI. As depicted in Figure S12a (Supporting Information), the cell viability of LO2 cells co‐incubated with SPI for 24 h was ≈1.25‐fold higher compared to untreated LO2 cells. Moreover, it has been reported that the enzymatic peptides derived from SPI possess the capability to effectively eliminate ROS.^[^ ^28^ ^]^ As presented in Figure S12b (Supporting Information), the exposure to H_2_O_2_ not only triggered the excessive generation of intracellular ROS but also caused cell death. Nonetheless, the addition of SPI‐derived peptides, produced via the hydrolysis of NPr at 45 °C, significantly mitigated the damage caused by ROS overaccumulation. The results showcased the immense potential of SPI in the field of tissue engineering, rendering it a fitting choice for integration into our responsive MN arrays. However, SPI, as a natural protein aggregate composed of highly ordered globulin subunits, possesses a compact molecular structure that imparts resistance against enzymatic hydrolysis.^[^ ^29^ ^]^ To address this, before incorporating SPI into the MNs, we heated the SPI solution in a water bath at 95 °C for 30 min (**Figure** **4**a), aiming to sufficiently unfold the molecular structures of SPI and expose more active sites. To prevent the refolding of SPI molecular structure or intermolecular cross‐linking leading to hydrogel formation, a biocompatible linear polymer, PVA, was added into the SPI solution prior to heating (Figure 4a).^[^ ^30^ ^]^ Following a gradual cooling process at room temperature, the mixed solution was promptly transferred into a polydimethylsiloxane (PDMS) mold to fabricate MN arrays through repeated vacuum‐assisted procedures (Figure S13a, Supporting Information). The resulting MN patch comprised a 15 × 15 array of SPI‐PVA needle tips containing NPr, SecNPs, and/or PtNZs, on the 8 mm × 8 mm sodium hyaluronate (SH) substrate (Figure 4a).

The morphology of the MNs was observed in Figure 4b,c,f, revealing needle tips with a characteristic rectangular pyramid shape. These needle tips exhibited a vertical height of ≈600 µm, a base width of ≈200 µm, and a needle pitch of ≈500 µm, closely matching the factory configuration of the PDMS molds employed. Besides, as depicted in Figure 4c, the MNs exhibited a distinct red fluorescence indicative of DiI‐labled SecNP incorporation, confirming the drug‐loading capability of our engineered MN arrays. The mechanical strength of MNs was evaluated through compression tests (Figure S13b, Supporting Information), and the representative stress‐strain curves for this evaluation were presented in Figure 4d. It was observed that the Young's modulus of MNs without NPr was ≈2.1 MPa, and the inclusion of NPr did not significantly affect this value (Figure 4e). Furthermore, the introduction of SecNPs or PtNZs also had no substantial impact on the moduli (Figure 4e). The morphology of MNs before and after the compression test is shown in Figure 4f. It was evident that the SPI‐PVA needle tips exhibited remarkable toughness, as they merely underwent bending without any fractures upon compression, thereby ensuring their seamless insertion into the liver tissue (Figure 4g).

### 2.8 In Vitro Photothermal Conversion Effect of MN Arrays

The photothermal effect of PtNZs encapsulated in MN (PMN) arrays was investigated following the method for evaluating the photothermal conversion capability of PtNZs. As anticipated, PMN arrays displayed a NIR power‐dependent temperature increase trend, ranging from ≈30 °C at 0.5 W cm^−2^ to ≈60 °C at 2 W cm^−2^ (Figure S14a, Supporting Information). Upon exposure to NIR irradiation at a power density of 1 W cm^−2^ for 10 min, the temperature of PMN arrays gradually rose, eventually stabilizing at a level determined by the concentration of PtNZs (Figure 4h; Figure S14b, Supporting Information). Considering the safe temperature range for mice and the optimal temperature for NPr activation, a concentration of 300 µg mL^−1^ (mass concentration in the SPI‐PVA solutions before filling and drying) was selected for PtNZs to achieve a final stable temperature ≈45 °C when incorporated into MNs for subsequent experiments. It is noteworthy that the concentration of PtNZs loaded in MNs was higher than that used in the aforementioned experiments involving intracellular ROS depletion and O_2_ generation (less than 10 µg mL^−1^). This was because the degradation of needle tips was both gradual and temperature‐responsive, resulting in a slow release of PtNZs over time, which would likely be excreted or degraded in vivo, thus limiting their in‐body presence during application. Furthermore, PMN arrays exhibited remarkable stability in terms of photothermal conversion undergoing three on/off cycles of NIR irradiation (Figure 4i).

Then, the NIR‐triggered temperature‐responsive drug release behavior of our smart MN arrays was evaluated. Under normal conditions, the cargos were slowly released over 1 h. However, when heated at 45 °C for 5 min, mimicking the NIR‐triggered temperature increase, the release rate increased dramatically. In contrast, the MN arrays not subjected to heating exhibited a consistent and lower drug release rate throughout, with the final cumulative drug release percentage remaining ≈20% lower than that of the heated MN arrays. The mechanism of NIR‐triggered NPr‐induced MN degradation and subsequent cargo release is elucidated in Figure 4k. Schematically, PtNZs exhibited the photothermal conversion capability upon NIR irradiation, activating the temperature‐sensitive protease NPr (with its optimum working temperature range of 45–55 °C) to hydrolyze SPI (the main component in MNs). The controlled degradation of MNs under NIR stimulation enables the responsive release of incorporated SecNPs and PtNZ into fibrotic liver tissues, facilitating the desired therapeutic effects.

### 2.9 In Vivo Antifibrotic Effects of MN Arrays

**Figure 5:**
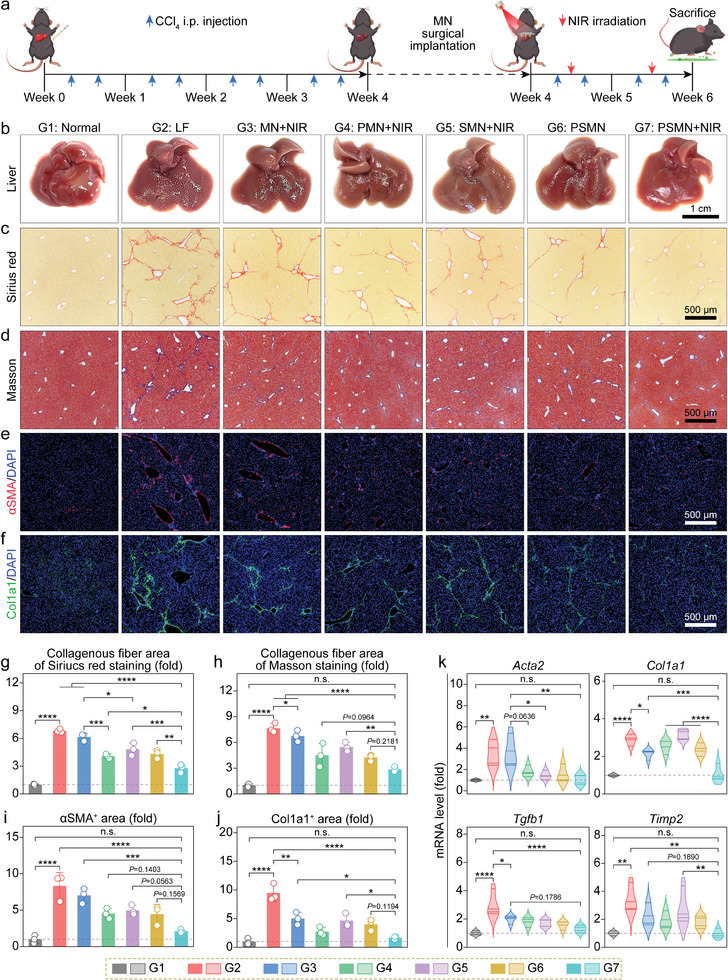
Rejuvenating effects of MN arrays in the liver‐fibrotic murine model. a) Schematic diagram illustrating the animal experimental procedures, including liver fibrosis murine model constructed by CCl_4_ intraperitoneal (i.p.) injection, treatment via in situ MN implantation followed by NIR irradiation, and sacrifice for sample harvest. b–f) Representative images for appearance (b), Sirius red staining (c), Masson's trichrome staining (d), αSMA immunofluorescence (e), and Col1a1 immunofluorescence (f) of liver tissue sections from mice undergoing the indicated treatments. g–j) Relative quantifications of Sirius red indicated fiber area (g), Masson's trichrome‐indicated fiber area (h), αSMA^+^ area (i), and Col1a1^+^ area (j) were performed based on the corresponding micrographs. k) Relative intrahepatic levels of mRNA implicated in liver fibrosis (*Acta2*, *Col1a1*, *Tgfb1*, and *Timp2*). Data are shown as mean ± SD (*n* = 3, panels g–j; *n* = 4, panel k). Statistical significances were evaluated with one‐way ANOVA followed by Tukey's multiple comparisons post hoc test. ^*^ *p* < 0.05; ^*^ *p* < 0.01; ^***^ *p* < 0.001; ^****^ *p* < 0.0001; and n.s., not significant.

Having confirmed the therapeutic efficacy of the cargos loaded within MNs through the in vitro experiments, we proceeded to investigate the antifibrotic effect of the smart MN arrays in a liver fibrosis animal model. The methodology for establishing a murine model of liver fibrosis and implementing in situ MN patch implantation followed by NIR irradiation are illustrated in **Figure** **5**a. Specifically, C57BL/6 mice received the intraperitoneal (i.p.) injection of CCl_4_ (12.5%, v/v) twice per week for 6 weeks to induce liver fibrosis.^[^ ^31^ ^]^ At the end of week 4, MN patches were implanted, and a two‐week treatment was administered. Initially, the mice were randomly divided into seven groups based on the therapeutic approaches: G1, normal group; G2, liver fibrosis (LF) model group without treatment; G3, treatment through blank MN patch implantation and NIR irradiation (MN+NIR); G4, treatment through PtNZ‐loaded MN (PMN) patch implantation and NIR irradiation (PMN+NIR); G5, treatment through SecNP‐loaded MN (SMN) patch implantation and NIR irradiation (SMN+NIR); G6, treatment through PtNZ+SecNP‐loaded MN (PSMN) patch implantation (PSMN); G7: treatment through PSMN patch implantation and NIR irradiation (PSMN+NIR). During the MN patch implantation surgery, the MN arrays were precisely inserted into the left lateral lobe of the liver in each mouse with liver fibrosis (Figure S15a, Supporting Information). Subsequently, a weekly exposure to 808 nm NIR irradiation for 5 min was employed to achieve localized heating (≈45 °C) at the implantation site (Figure S15b, Supporting Information).

The representative macroscopic graphs of livers from mice with different treatments are presented in Figure 5b. Consistent with the characteristic pathological features of liver fibrosis, the liver from the LF group exhibited a rough, uneven appearance with a darker color compared to the normal group, indicating the successful establishment of the animal model. In contrast, the liver from MN+NIR group displayed visually alleviated fibrosis and a slightly glossy appearance, possibly attributed to the ROS depletion caused by the active peptides produced by the gradual degradation of SPI.^[^ ^28^ ^]^ The livers from groups treated with MN arrays containing either PtNZs (PMN+NIR) or SecNPs (SMN+NIR) demonstrated more pronounced relief in fibrosis, which was induced by the respective therapeutic effects validated at the cellular level. Although the livers from groups treated with dual‐drug MN arrays (PSMN and PSMN+NIR) both exhibited distinct fibrosis mitigation, the liver from PSMN+NIR group was particularly smoother, glossier, and had a healthier ruddy appearance. This enhanced effect could be attributed to the photothermal activity of the PtNZs in the PSMNs under NIR irradiation, which activated NPr, leading to the hydrolysis of SPI and the subsequent degradation of MNs to release the therapeutic PtNZs and SecNPs. Contrastively, the PSMNs exhibited a decelerated degradation in the absence of NIR irradiation, leading to compromised therapeutic efficacy.

Additionally, histochemical analyses were conducted on the collected liver tissues. The Sirius red‐stained (Figure 5c) and Masson's trichrome‐stained (Figure 5d) liver sections demonstrated consistent trends with the visual observations The fibrosis area (red for Sirius red staining; blue for Masson's trichrome staining) in the PSMN+NIR group was significantly smaller compared to other groups undergoing CCl_4_ injection, constituting ≈35% of that observed in the LF group (Figure 5g,h). Notably, the quantification of Masson's trichrome‐staining images (Figure 5h) revealed that the liver fibrosis level in PSMN+NIR group closely resembled that of the normal group. The immunofluorescence (IF) stained liver sections reflected a significant upregulation of fibrosis markers αSMA (Figure 5e,i) and Col1a1 (Figure 5f,j) in the LF group, indicating HSC activation and ECM deposition. The treatments with MN arrays significantly reduced the intrahepatic distribution areas of αSMA (Figure 5e,i) and Col1a1 (Figure 5f,j), with the PSMN+NIR group exhibiting the most substantial decrease, amounting to ≈80% of those in the LF group and approaching the levels found in the normal group (Figure 5i,j). Furthermore, the levels of intrahepatic mRNA for representative profibrotic factors were assessed using RT‐qPCR assays to evaluate the therapeutic effects. As depicted in Figure 5k, compared to the LF group, liver tissues from the PSMN+NIR group exhibited significantly diminished mRNA levels of *Acta2* (by ≈85%), *Col1a1* (by ≈75%), *Tgfb1* (by ≈75%), and *Timp2* (by ≈80%), closely resembling those observed in the normal group. Therefore, our implementation of a comprehensive therapeutic approach using responsive PSMN arrays and NIR irradiation effectively alleviated liver fibrosis.

### 2.10 In Vivo Anti‐Inflammatory and Hepatoprotective Effects of MN Arrays

**Figure 6:**
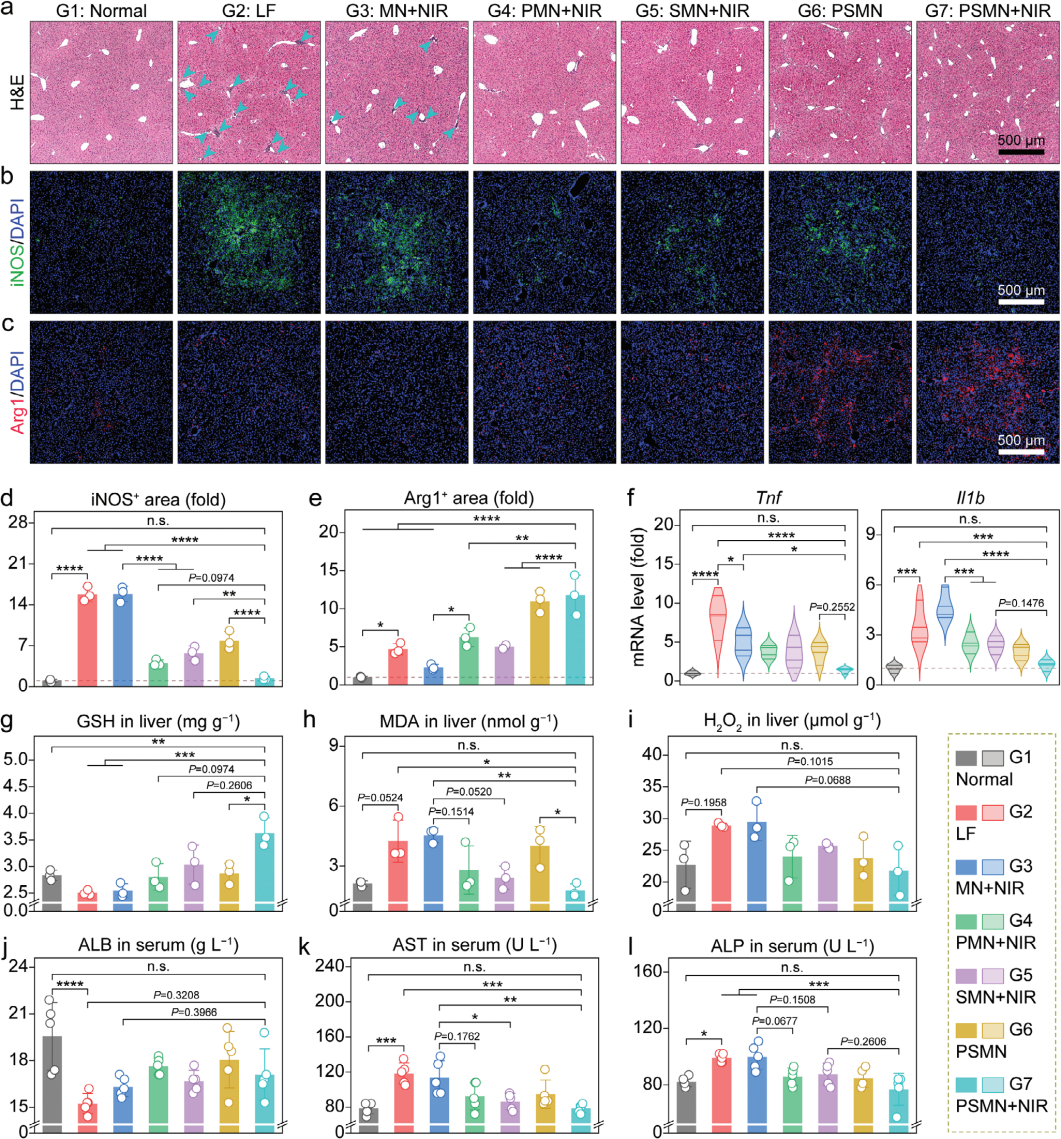
Anti‐inflammatory and hepatoprotective effects of MN arrays in the liver‐fibrotic murine model. a–c) Representative images for H&E staining (arrows indicate immune cell infiltration) (a), iNOS immunofluorescence (b), and Arg1 immunofluorescence (c) of liver tissue sections from mice following the indicated treatments. d,e) Relative quantifications of iNOS^+^ area (d), and Arg1^+^ area (e) conducted based on the corresponding micrographs. f) Relative intrahepatic mRNA levels of proinflammatory factors (*Tnf* and *Il1b*). g–i) Intrahepatic levels of factors involved in redox homeostasis and oxidative stress, encompass GSH (g), MDA (h), and H_2_O_2_ (i). j–l) Serum levels of factors associated with liver function and dysfunction, including ALB (j), AST (k), and ALP (l). Data are presented as mean ± SD (*n* = 3, panels d, e, and g–i; *n* = 4, panel f; *n* = 5, panels j–l). Statistical significances were estimated employing one‐way ANOVA with Tukey's multiple comparisons post hoc test. ^*^ *p* < 0.05; ^**^ *p* < 0.01; ^***^ *p* < 0.001; ^****^ *p* < 0.0001; and n.s., not significant.

The presence of chronic inflammation and hepatocellular damage represents typical complications associated with liver fibrosis, thus necessitating an evaluation of the anti‐inflammatory and hepatoprotective effects exhibited by our responsive MN arrays.^[^ ^32^ ^]^ The H&E‐stained liver sections displayed a remarkable infiltration of immunocytes (primarily monocytes and neutrophils), indicating an inflammatory response, as well as disrupted hepatocyte arrangement in the livers from the LF group (**Figure** **6**a). However, the treatment with PSMN+NIR showed a noticeable regression in immunocyte infiltration and restored normal hepatocyte morphology and arrangement. Then, the phenotypic markers of proinflammatory M1 (iNOS, Figure 6b) and pro‐reparative M2 (Arg1, Figure 6c) macrophages were subjected to IF staining. As presented in Figure 6d, in comparison to the normal group, the LF group exhibited a remarkable ≈15‐fold increase in the iNOS‐positive area, reflecting the occurrence of the inflammation accompanying liver fibrosis. Post‐treatment with PSMN+NIR, the iNOS expression nearly vanished within liver tissues, reverting to the levels comparable to those observed in the normal group and thereby demonstrating effective elimination of inflammation. Additionally, the Arg1‐positive area of liver tissue from the PSMN+NIR group was the largest (Figure 6e), surpassing 11 times larger than that found in the normal group. This signified the successful initiation of M2‐mediated phagocytosis of hepatocyte debris and subsequent tissue repair facilitated by the in situ PSMN patch implantation and localized NIR irradiation. In addition to the histological evaluation, the intrahepatic mRNA abundances of representative proinflammatory factors were also investigated (Figure 6f), with the results in line with IF staining for iNOS and revealing that the comprehensive intervention of PSMN patches and NIR distinctly downregulated the expressions of *Tnf* (declining by ≈90% compared to the level in the LF group) and *Il1b* (decreasing by ≈70% than the level in the LF group) in liver tissues.

After the confirmation of in vivo anti‐inflammatory function, the hepatoprotective effect of our designed NIR‐responsive MN arrays was also evaluated. First, the IF staining for Ki67 (indicating proliferation) and terminal deoxynucleoitidyl transferase‐mediated deoxyuridine triphosphate nick end labeling (TUNEL, reflecting apoptosis) assays were separately performed on liver tissues from the normal, LF, and PSMN+NIR groups. As illustrated in Figure S16 (Supporting Information), the continuous i.p. injection of CCl_4_ indeed suppressed the intrahepatic proliferation and induced apoptosis. The fibrosis‐associated hepatic damage was reversed by the treatment with PSMN+NIR. Reduced glutathione (GSH), an intracellular antioxidant, plays a pivotal role in hepatoprotection.^[^ ^33^ ^]^ As illustrated in Figure 6g, the GSH level was found to be reduced in the fibrotic liver but significantly increased in the PSMN+NIR group, exhibiting an ≈1.5‐fold elevation compared to that observed in the LF group. Malondialdehyde (MDA), a typical byproduct of lipid peroxidation in the liver, exhibits severe cytotoxicity due to its capacity to denature intracellular nucleic acids and proteins.^[^ ^34^ ^]^ Therefore, the MDA levels can generally reflect the degree of liver injury. To determine the MDA levels in liver tissue homogenates from different groups, an MDA assay kit was employed. The result showed that the MDA content in the liver tissues from the LF group was about two‐fold higher than that in the normal group, whereas PSMN+NIR treatment significantly reduced the MDA content in liver tissues, aligning them with levels observed in healthy mice (Figure 6h).

The H_2_O_2_ level in liver homogenates was also measured, and the results showed an obvious decline of ≈30% in the PSMN+NIR group compared to that found in the LF group (Figure 6i). The secretion of albumin (ALB), a secreted protein, can be inhibited by liver injury, including liver fibrosis.^[^ ^12^, ^35^ ^]^ As depicted in Figure 6j, the ALB content in the serum was nearly restored to normal levels, following the treatment with PSMN+NIR. Aspartate transaminase (AST) and alkaline phosphatase (ALP) are the two prominent endogenous enzymes in the liver, and their release into the peripheral blood usually means liver injury.^[^ ^36^ ^]^ The successful induction of liver fibrosis resulted in significant increases in AST levels (≈1.5‐fold higher than the healthy level, Figure 6k) and ALP levels (≈1.2‐fold higher compared to the healthy level, Figure 6l) in the serum. However, the treatment with PSMN patch implantation combined with NIR irradiation effectively reduced these enzyme levels in the serum, restoring them to healthy levels in the normal group.

Besides, a reduction in the abnormal total bilirubin (TBIL) elevation caused by liver fibrosis was evident in the PSMN+NIR group (Figure S17a, Supporting Information). The other blood biochemical indices, including blood urea nitrogen (BUN, reflecting the kidney function), creatinine (CREA, indicating the kidney function), creatine kinase (CK, indicating the heart function), and lactate dehydrogenase (LDH, reflecting the heart function), did not show any significant differences between the treatment groups and the normal group or fall outside the corresponding recommended ranges for all the seven groups, suggesting no significant impact on kidney or heart functions (Figure S17b–e, Supporting Information).^[^ ^37^ ^]^ Furthermore, no pathological abnormalities or variations were found in the representative H&E‐staining images of the heart (Figure S18, Supporting Information), spleen (Figure S19, Supporting Information), lung (Figure S20, Supporting Information), and kidney (Figure S21, Supporting Information) from the seven groups. These findings unequivocally demonstrated the decent histocompatibility and biosafety of our engineered MN arrays and performed therapeutic procedures. Collectively, the in situ implantation of the responsive MN arrays incorporating PtNZs and SecNPs, combined with the targeted NIR irradiation, demonstrated superior therapeutic efficacy against liver fibrosis. This efficacy was mainly manifested through the remarkable antifibrotic, anti‐inflammatory, and antioxidative effects, as well as the restoration of liver function.

### 2.11 Transcriptome Analysis

**Figure 7:**
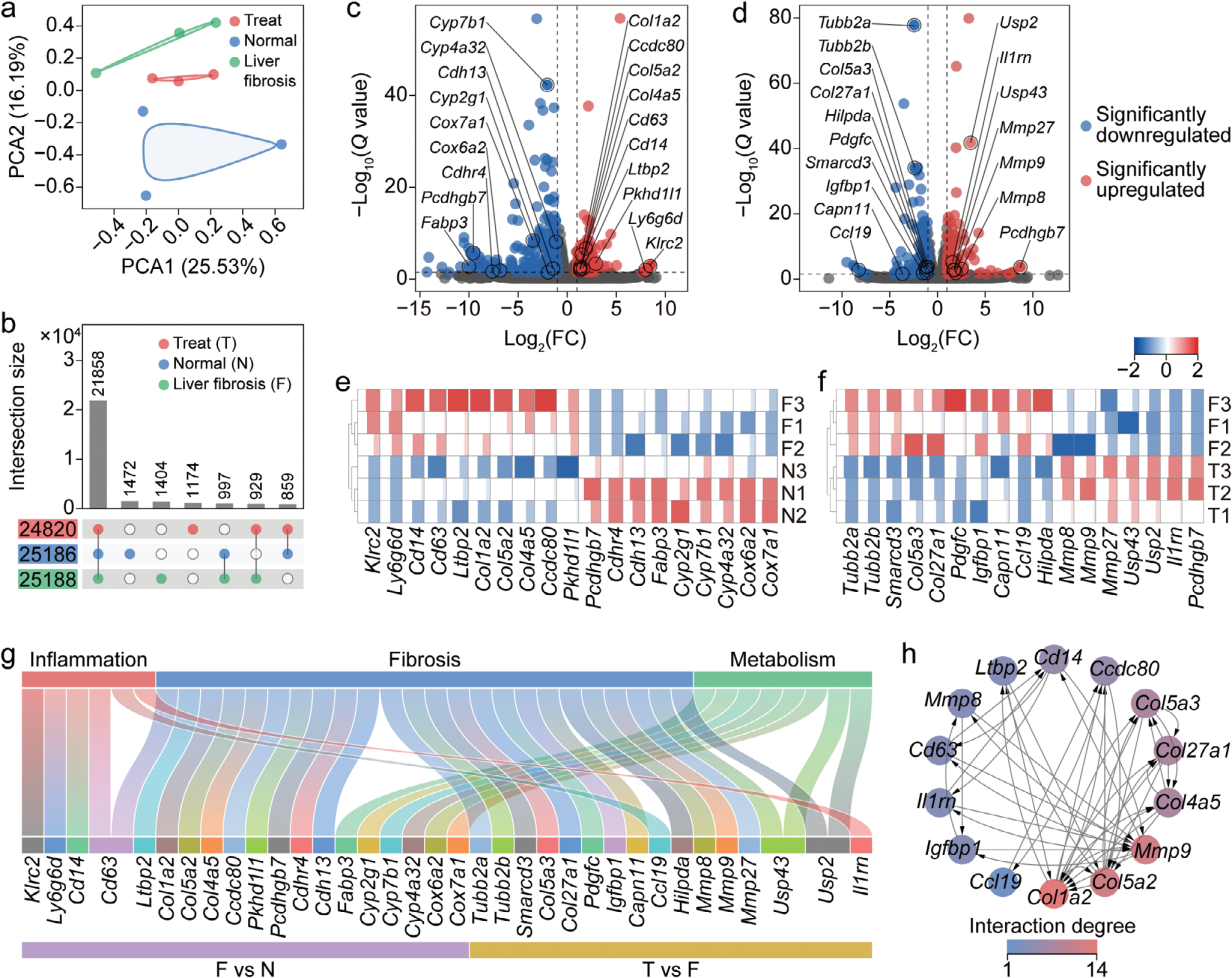
Transcriptome analysis based on RNA sequencing of liver tissues from LF, normal, and PSMN+NIR groups. a,b) Similarities, dissimilarities, and gene count among all tested liver samples depicted by PCA scatter plot (a) and upset plot (b). c,d) Volcano plots illustrating significantly upregulated (*Q* < 0.05 and |log_2_FC| > 1) and significantly downregulated (*Q* < 0.05 and |log_2_FC| < 1) genes and the GOIs in DEGs between LF (F) and normal (N) groups (c), and between PSMN+NIR (treatment, T) and F groups (d). *Q* value = adjusted *P* value. e,f) Heatmaps displaying expression variations of GOIs between F group and N group (e), and between F group and T group (f). g) Sankey diagram with pathway enrichments of all GOIs among F, N, and T groups. h) PPI network of GOIs among F, N, and T groups.

To further bolster the in vitro and in vivo experimental findings and authenticate the inferred therapeutic mechanisms, a comprehensive analysis of total transcriptome RNA sequencing was conducted on nine randomly selected liver samples, encompassing three each from the normal (N), LF (F), and PSMN+NIR (treatment, T) groups. A principal component analysis (PCA) was first performed based on the gene expression matrix, revealing distinct transcriptomic separations among the N, F, and T groups along the first principal component (PC1) (**Figure** **7**a). This visualization effectively illustrated significant disparities in gene expression among the three experimental groups. The higher‐order upset plot indicated a remarkable co‐expression of 21 858 genes across the three groups (Figure 7b), with 997 genes exclusively co‐expressed in the F and N groups, and 929 genes in the T and F groups. The differentially expressed genes (DEGs) were defined according to their statistical significance at a level of *Q* < 0.05 (*Q* value = adjusted *P* value) and fold change (FC) criterion with |log_2_FC| > 1. The heatmaps showed weak intergroup correlations but stronger intragroup correlations among the samples in the F and N groups (Figure S22a, Supporting Information) or the T and F groups (Figure S22b, Supporting Information). The volcano plots in Figure 7c and the heatmap in Figure S22a (Supporting Information) co‐revealed that, compared to the N group, 148 significantly upregulated (*Q* < 0.05 and log_2_FC > 1) genes and 196 significantly downregulated (*Q* < 0.05 and log_2_FC < −1) genes were observed in the F group.

Additionally, the volcano plot in Figure 7d and the heatmap in Figure S22b (Supporting Information) demonstrated the coexistence of 133 significantly upregulated (*Q* < 0.05 and log_2_FC > 1) genes and 176 significantly downregulated (*Q* < 0.05 and log_2_FC < −1) genes in the T group, compared to the F group. Based on these findings, we meticulously selected 19 genes of interest (GOIs) (Figure 7c,e) and 17 GOIs (Figure 7d,f) from the DEGs identified between the F and N groups, as well as between the T and F groups. Subsequently, two heatmaps (Figure 7e,h) were separately generated to visually represent the intricate expression variations of these GOIs. All the GOIs were implicated in the underlying mechanisms of inflammation facilitation (in conjunction with liver fibrosis progression) and inhibition (via PSMN+NIR treatment), HSC activation (liver fibrosis) and quiescence (PSMN+NIR treatment), ECM deposition (liver fibrosis) and degradation (PSMN+NIR treatment), as well as liver metabolism regulation (Figure 7g). Furthermore, the protein–protein interaction (PPI) network analysis based on the STRING database and GOIs provided insights into the functional relationships among key proteins in liver fibrosis, such as Col1a2, Col5a2, MMP9, Col4aa, Col27a1, and Col5a3 (Figure 7h). These crucial factors exhibited extensive interactions with proteins encoded by other GOIs among the T, N, and F groups and possessed direct regulatory capabilities over ECM deposition or degradation.

**Figure 8:**
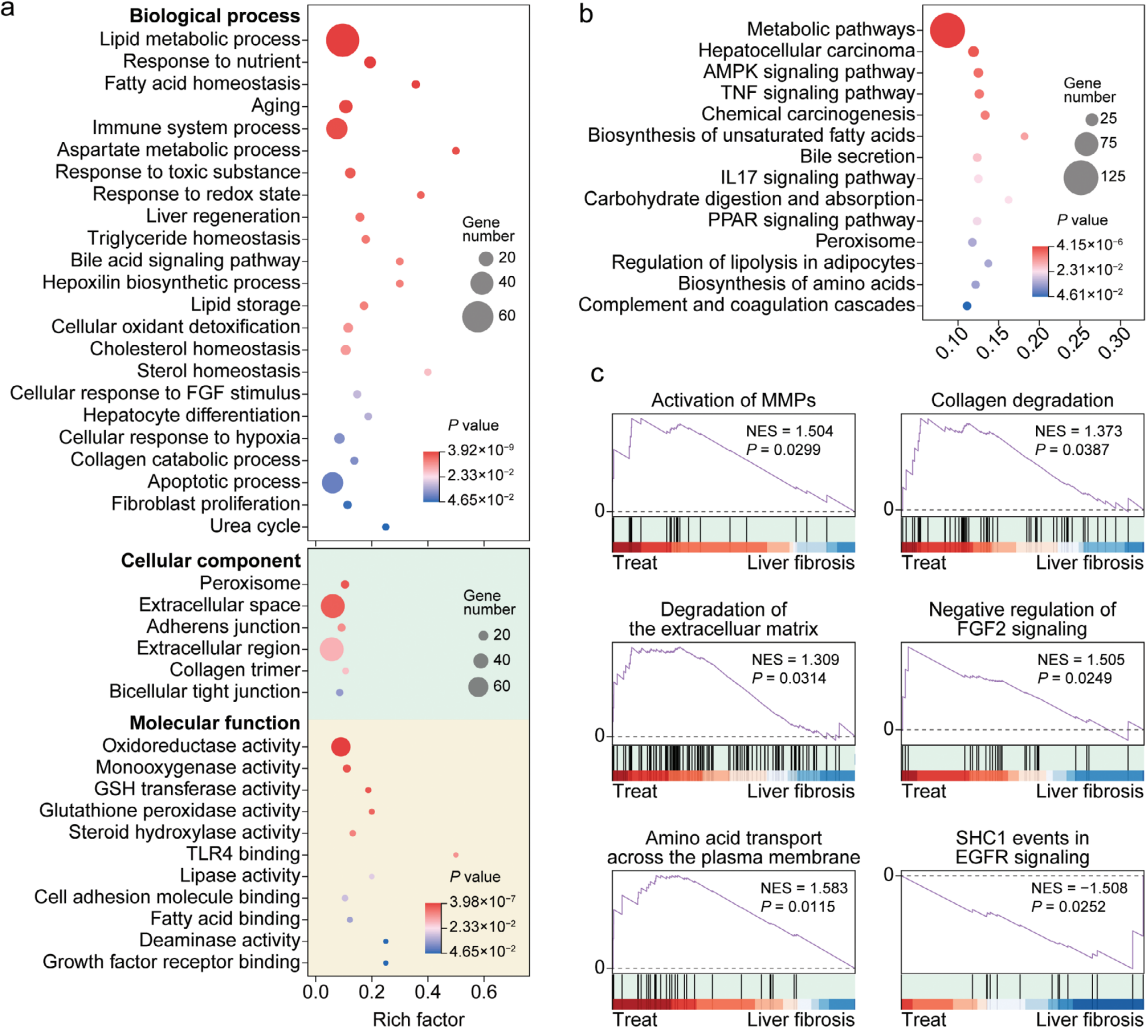
Transcriptome enrichment analysis revealing mechanisms of the liver fibrosis treatment with PSMN+NIR. a,b) Enrichments of DEGs in liver samples from PSMN+NIR group and LF group in various GO terms (a) and KEGG pathways (b). c) GSEA based on Reactome enrichments indicating upregulated (*p* < 0.05 and NES > 1) and downregulated (*p* < 0.05 and NES < –1) pathways involved in ECM deposition and degradation, as well as HSC activation and quiescence.

To gain deeper insights into the underlying mechanisms of the liver fibrosis treatment with PSMN+NIR, the enrichment analyses of gene ontology (GO) terms (Figures S23 and S25, Supporting Information; **Figure** **8**a) and Kyoto encyclopedia of genes and genomes (KEGG) pathways (Figures S24 and S26, Supporting Information; Figure 8b) were performed on the DEGs between the F and N groups (Figures S23 and S24, Supporting Information), as well as between the T and F groups (Figure 8a,b; Figures S25 and S26, Supporting Information). As presented in Figure 8a, the GO terms were significantly enriched in the biological processes involving lipid metabolic process response to nutrient, fatty acid homeostasis, and immune system process; cellular components including peroxisome, extracellular space, adherence junction, and collagen region; and molecular functions encompassing oxidoreductase activity, monooxygenase activity, GSH transferase activity, and glutathione peroxidase activity. The KEGG pathways shown in Figure 8b exhibited remarkable enrichment in metabolic pathways, hepatocellular carcinoma, AMPK signaling pathway, TNF signaling pathway, and chemical carcinogenesis. All the mentioned GO terms and KEGG pathways were closely associated with inflammation suppression, fibrosis remission, and metabolism regulation in the fibrotic liver treated with PSMN+NIR.

Furthermore, the gene set enrichment analysis (GSEA) on the DEGs between the F and N groups, and between the T and F groups were also carried out. The results of the GSEA on DEGs between the F and N groups were presented in Figure S27 (Supporting Information), indicating that the continuous i.p. injection of CCl_4_ indeed upregulated the intrahepatic crosslinking of collagen fibrils, heparan sulfate‐glycosaminoglycan biosynthesis, integrin cell‐surface interactions, and formation of senescence‐associated heterochromatin foci, while downregulating neddylation and synthesis of very‐long‐chain fatty acyl‐CoAs. The results of the GSEA on DEGs between the T and F groups were shown in Figure 8c, unequivocally revealing that, compared to the F group, the treatment with PSMN+NIR could upregulate activation of MMPs, collagen degradation, ECM degradation, negative regulation of FGF2 signaling, and amino acid transport across the plasma membrane, as well as downregulate SHC1 events in EGFR signaling associated with HSC activation. In summary, the transcriptome analysis based on RNA sequencing of liver tissues from the normal, LF, and PSMN+NIR groups not only reaffirmed the triumphant establishment of a CCl_4_‐induced liver fibrosis model but also yielded profound revelations regarding the therapeutic effectiveness and underlying mechanisms of PSMN+NIR in treating liver fibrosis.

## 3 Discussion

Liver fibrosis is a chronic pathological process characterized by intrahepatic excessive ECM deposition due to persistent liver injuries, for which there is currently no specific clinical intervention available. MSCs have been extensively employed in tissue regeneration and repair, primarily owing to their robust paracrine effect, which has demonstrated remarkable efficacy. Although MSC therapy has shown promising potential in treating liver diseases, its practical application is hindered by various obstacles including potential immune rejection, cancer risk, unstable efficacy, and high treatment costs.^[^ ^7^ ^]^ Instead, the MSC secretome encompasses a diverse range of bioactive substances, showcasing immense potential as cell‐free therapeutic agents. However, during liver fibrosis development, the perisinusoidal space undergoes substitution with dense ECM and collagen scar tissue, which function as formidable barriers severely impeding drug delivery. Meanwhile, traditional injection methods face challenges related to low targeting specificity, limited delivery efficiency, and inadequate retention rate.^[^ ^14^ ^]^ Although the in situ use of hydrogel patches or scaffolds also allows for continuous targeted drug delivery to fibrotic liver tissues, it is important to note that therapeutic agents released on the surface of the liver capsule are susceptible to clearance. Furthermore, their effective diffusion into the liver parenchyma is hindered by traversing through the liver capsule. The MN array systems stand out as the only method capable of directly delivering therapeutic agents through the liver capsule to reach the liver parenchyma, thereby overcoming challenges such as rapid clearance, high depletion, and off‐target effects. Therefore, this study aims to address these crucial problems by offering novel breakthroughs and solutions based on smart MN delivery techniques to treat liver fibrosis.

In previous studies, researchers have combined stem cell therapy with MN delivery to facilitate in situ repair after spinal cord injury.^[^ ^38^ ^]^ Furthermore, MN array systems have been used by researchers to achieve the repair and regeneration of cardiac tissue in animal models of myocardial infarction and ischemic heart disease.^[^ ^39^ ^]^ Building upon the potential of MN delivery techniques for tissue engineering and regenerative medicine, we developed an implantable smart MN array system synchronously performing cell‐free therapy and platinum‐based nanocatalytic therapy. The smart MNs were constructed using responsively hydrolyzable SPI and biocompatible PVA, loaded with PtNZs, SecNPs, and NPr, possessing the capability to perforate the liver capsule and deliver multiple therapeutic agents directly into the fibrotic liver tissue in a precisely controlled manner. Notably, PtNZs in the MNs exhibited a remarkable photothermal effect under NIR irradiation. By modulating the power intensity and exposure duration of NIR irradiation, the temperature at the implantation site was elevated to the optimal working range of NPr, thereby effectively activating SPI hydrolysis. This NIR‐responsive degradation enabled the gradual release of PtNZ and SecNPs into the fibrotic liver tissue, achieving a sustained therapeutic effect. The released SecNPs were instrumental in deactivating HSCs, reducing ECM deposition, enhancing ECM degradation, protecting hepatocytes from damage, promoting hepatocyte proliferation, and mitigating inflammation. Concurrently, the released PtNZs could efficiently neutralize excessive ROS and alleviate hypoxia, a common complication of fibrosis progression.

In summary, our innovative approach integrating the in situ implantation of smart PSMN arrays and localized NIR irradiation demonstrated significant potential in alleviating liver fibrosis, showing promising avenues for clinical application. However, in the investigation of the antifibrotic effect of SeCNPs, we primarily presented compelling evidence for its functionality without delving into the underlying molecular biological mechanism. In order to acquire a more comprehensive understanding of how SeCNPs exert their antifibrotic effect, future studies should be conducted systematically and in‐depth. Additionally, there is a potential risk of invasive and postoperative infection associated with surgical implantation. Therefore, in future developments, we will aim to implement MN patches using minimally invasive methods. Although our developed PSMN has shown promising outcomes at the cellular and animal levels, it is evident that significant advancements are necessary before the translation to human applications. Further investigation and rigorous research are imperative regarding the dimensions of the PSMN patch suitable for the human liver, drug‐carrying capacity, and mitigating surgical risks. In our upcoming research endeavor, we will focus on refining this delivery system to offer a reliable and efficient treatment alternative for liver fibrosis.

## 4 Experimental Section

### Collection and Purification of Secretome

The hUCMSC‐derived secretome was generously provided by the Biotherapy Center (the Third Affiliated Hospital, Sun Yat‐sen University, Guangzhou, China). The isolation procedure for hUCMSCs was approved by the Research Ethics Committee at the Third Affiliated Hospital of Sun Yat‐sen University (approval number: 2022‐02‐062). Informed consent was obtained from all participants or their legal guardians in accordance with ethical guidelines established by the hospital. After obtaining informed consent from parturients, fresh umbilical cords were collected to acquire hUCMSCs. Following purification through passaging, once they reached 70–80% confluence, the hUCMSCs were washed twice with PBS and then subjected to an additional 48‐h incubation period in nonsupplemented medium. The serum‐free medium was collected, followed by centrifugation and filtration through filters with an average pore diameter of 0.22 µm to eliminate any potential cells and cell debris. The filtered conditioned medium was immediately frozen using liquid nitrogen and subsequently subjected to lyophilization. The desiccated secretome was then stored at −80 °C for further use. The protein components and abundances of the collected hUCMSC‐derived secretome were evaluated using the label‐free proteome analysis conducted by LC‐Bio Technology (Hangzhou, China).

### Fabrication and Characterization of SecNPs

The purified and lyophilized secretome powder (2 mg) was dissolved in ultrapure water (2 mL), as the aqueous phase. Simultaneously, PLGA (0.2 g) and Span 80 (0.01 g) were dissolved in DCM (10 mL), as the oil phase. The aqueous phase was then added into the oil phase and pre‐emulsified with a high‐speed shearing homogenizer (T18 digital, IKA, Germany) at 15 000 rpm for 5 min. The initial emulsification was performed with an ultrasonic homogenizer (950E, Scientz, China) operating at 300 W for 5 min with an on/off cycle of 3 s/3 s. Subsequently, the initial emulsion was dropwise introduced into a 2% (weight/volume, w/v) BSA solution (10 mL), followed by the re‐emulsification using an ultrasonic homogenizer operating at 100 W for 2 min (on/off = 3 s/3 s). Finally, the PVA (2%, w/v) solution (12 mL) was added to the resulting double emulsion. After continuous magnetic stirring for 24 h to allow complete evaporation of DCM, the SecNPs were obtained through centrifugation at 12 000 rpm for 15 min, followed by three washing steps and subsequent storage at 4 °C.

The hydrodynamic diameter and zeta potential of the fabricated SecNPs were measured using DLS and ELS techniques, respectively, with a nanoparticle analyzer (Litesizer 500, Anton‐Paar, Austria). A TEM (Tecnai G2 Spirit, FEI, USA) instrument was employed to visualize the microtopography of SecNPs.

### RNA Extraction and RT‐qPCR

The total RNA was extracted from the cells or liver tissues using Trizol reagents (Cowin, China). The RNA concentration and purity were determined using a UV–vis ultramicro‐spectrophotometer (NanoDrop2000, Thermo). The cDNA was synthesized employing a reverse transcription kit (PreScript III, EnzyValley, China) and a PCR thermocycling instrument (ProFlexTM Base, Thermo). Subsequently, RT‐qPCR assays based on the synthesized cDNA and designed primers (Table S1, Supporting Information) were conducted with a 2× robust SYBR Green kit (EnzyValley) and an RT‐qPCR instrument (QuantStudio 5, Thermo). The 2^Ct^ (Ct = amplification cycle threshold) value of each quantified gene in each sample was normalized to that of *Gapdh* (murine or rat sample) or *GAPDH* (human sample) as an internal reference, and the corresponding mRNA levels were expressed as a fold change relative to the control group. The primers (Table S1, Supporting Information) were synthesized by Tsingke Biotech (Beijing, China).

### Synthesis of PtNZs

The PtNZs were synthesized by reducing H_2_PtCl_6_ (300 µm) with dimethylamine borane (DMAB) in a BSA (10 µm) solution (pH 4.0). After incubation at room temperature for 2 h, the mixture was supplemented with DMAB (1.5 mm) to initiate the reduction reaction. Following a minimum of 5 h of reaction time, the solution underwent a distinct color transformation to a clarified black‐brown hue, indicating the successful synthesis of PtNZs.

The hydrodynamic diameter and zeta potential were characterized using a nanoparticle analyzer. The absorption spectrum was determined by employing a UV–vis spectrophotometer (UV‐2600, Shimadzu, Japan). TEM analysis was conducted to visualize the size and morphology of PtNZs.

### Fabrication and Characterization of MN Patches

The SPI solution (3%, w/v) and PVA solution (1%, w/v) were combined in an aqueous medium, followed by heating at 95 °C for 30 min to induce protein denaturation, molecular unfolding, and subsequent hydration. Neutral protease (1 000 U g^−1^ SPI), SecNPs (900 µg), and PtNZs (900 µg) were thoroughly mixed and dispersed in the SPI+PVA solution (3 mL) using vortex and ultrasonication techniques in a sequential manner. Then, a detached MN patch was prepared according to previous reports with slight modifications.^[^ ^40^ ^]^ The mixture solution (100 µL) was dispensed into the PDMS microneedle mold (ST‐15×15‐H600B200P500, Micropoint Technologies, Singapore), and the cavities were progressively filled through repetitive vacuuming to form the needle tips of PSMN arrays. The redundant solution and bubbles were eliminated before overnight drying and subsequent solidification. The tips were covered with a substrate solution of 10% (w/v) SH (100 µL) followed by drying at room temperature for 24 h. Then, PSMN patches were yielded after the demolding process. Additionally, blank MN, PMN, and SMN patches were fabricated using a similar preparation procedure. All MN patches were stored under controlled dry conditions at 4 °C.

The brightfield and fluorescence microscopy images of MN patches were captured with a microscope (DS‐Ri2, Nikon, Japan). The microtopography of MNs before and after compression test was examined using scanning electron microscopy (SEM) equipment (Quanta 400 F, FEI, USA).

### Compression Test of MN Arrays

The mechanical strength of MN arrays was measured using an electronic universal material testing machine (5943, Instron, USA). Specifically, MN patches were placed on the stationary platform at the base of the apparatus. Then, the upper mobile station connected to a sensor, gradually approached the MN arrays at a constant speed of 0.2 mm s^−1^. The stress and displacement were continuously monitored in real time throughout the entire process. The compression strength was calculated according to the acquired stress and strain.

### Photothermal Effect of MN Arrays Containing PtNZs

The photothermal property of PMN arrays was determined. MN arrays with various concentrations of PtNZs (0, 150, 200, 250, 300, and 600 µg mL^−1^ in the preparing solution) were exposed to NIR irradiation at a constant power density of 1 W cm^−2^ using an 808 laser (Lasever, China). Additionally, MN arrays encapsulating PtNZs (300 µg mL^−1^) underwent 808 nm NIR irradiation at different power levels (0.5, 1, 1.5, 2 W cm^−2^). The temporal evolution of MN temperature was monitored and recorded with a thermal imaging camera (Hti HT‐19, Xintai, China).

### Animal Experiment

All animal studies were conducted in accordance with the ethical regulations and protocols approved (SYSU‐IACUC‐2023‐000357) by the institutional animal care and use committee at Sun Yat‐Sen University. For the CCl_4_‐induced liver fibrosis mouse model, male C57BL/6 mice aged 6–8 weeks received an i.p. injection of flaxseed oil containing 12.5% (v/v) CCl_4_ at a dosage of 5 µL g^−1^ twice weekly for 6 weeks. After 4 weeks of CCl_4_ injections, the liver fibrosis was preliminarily developed.^[^ ^31^ ^]^ At the end of week 4, one piece of MN patch was surgically implanted onto the left lateral lobe of the liver in each mouse with induced liver fibrosis (Figure S15a, Supporting Information). In detail, the surgical instruments were pre‐sterilized prior to use. Following this, the mice were anesthetized with sodium pentobarbital, and the abdominal skin was shaved. Subsequently, they were placed on a heat preservation pad (which had undergone advanced ultraviolet irradiation sterilization) in an ultra‐clean table and subjected to iodophor disinfection. The abdomen was then opened along the midline, exposing the liver. Careful positioning of a microneedle patch on the surface of the largest left lateral lobe of the liver was performed, followed by gentle pressing using forceps for ≈30 s to ensure proper penetration of needle tips into liver tissue. Before closing the abdomen, normal saline (100–300 µL) was instilled into the abdominal cavity for water replenishment using a syringe. Finally, absorbable sutures were utilized to close up the abdomen. During the subsequent two‐week treatment, the implantation site of mice was exposed to 808 nm NIR irradiation for 5 min (Figure S15b, Supporting Information) on a weekly basis, while CCl_4_ continued to be i.p. injected twice weekly. The initial NIR irradiation was performed on the third day following the surgical implantation. At the end of week 6, all mice were sacrificed, and the peripheral blood, liver, and other major organs were sampled and subjected to further experiments and analyses.

### Statistical Analysis

Data are presented as mean ± standard deviation (SD), with error bars indicating SD. The mean and SD values were calculated from three or more independent experiments. Statistical differences among three or more groups, with one nominal‐level variable, were assessed using one‐way analysis of variance (ANOVA) followed by Tukey's multiple comparisons post hoc test. Asterisks denoted different levels of significance (^*^ *p* < 0.05, ^**^ *p* < 0.01, ^***^ *p* < 0.001, and ^****^ *p* < 0.0001), while “n.s.” indicated nonsignificance.

## Conflict of Interest

The authors declare no conflict of interest.

## Supporting information

Supporting Information

## Data Availability

The data that support the findings of this study are available from the corresponding author upon reasonable request.
