# Supplementary material for: Smart Microneedle Arrays Integrating Cell‐Free Therapy and Nanocatalysis to Treat Liver Fibrosis
Source: Adv Sci (Weinh). 2024 Jun 14;11(31):2309940. doi: 10.1002/advs.202309940 (PMC11336984; doi:10.1002/advs.202309940)
Supplement: Supplementary file 1 — Supporting Information [file ADVS-11-2309940-s001.pdf]

ADVANCED SCIENCE Open Access

Supporting Information

for *Adv. Sci.*, DOI 10.1002/advs.202309940

Smart Microneedle Arrays Integrating Cell-Free Therapy and Nanocatalysis to Treat Liver Fibrosis

Yanteng Xu, Yixin Zhang, Hao Tian, Qingguo Zhong, Ke Yi, Fenfang Li, Tiantian Xue, Haixia Wang, Yeh-Hsing Lao, Yingying Xu, Yinxiong Li, Ling Long, Kai Li, Yu Tao* and Mingqiang Li**

Supporting Information

Smart Microneedle Arrays Integrating Cell-Free Therapy and Nanocatalysis to Treat Liver Fibrosis

Yanteng Xu, Yixin Zhang, Hao Tian, Qingguo Zhong, Ke Yi, Fenfang Li, Tiantian Xue, Haixia Wang, Yeh-Hsing Lao, Yingying Xu, Yinxiong Li, Ling Long, Kai Li, Yu Tao*, and Mingqiang Li**

Y. Xu, Y. Zhang, H. Tian, Q. Zhong, K. Yi, F. Li, T. Xue, H. Wang, K. Li, Y. Tao, M. Li
Laboratory of Biomaterials and Translational Medicine
Center for Nanomedicine and Department of Ultrasound
The Third Affiliated Hospital
Sun Yat-sen University
Guangzhou 510630, China
E-mail: likai@mail.sysu.edu.cn; taoy28@mail.sysu.edu.cn; limq567@mail.sysu.edu.cn

H. Tian, L. Long
Department of Neurology
The Third Affiliated Hospital
Sun Yat-sen University
Guangzhou 510630, China

Y.-H. Lao
Department of Pharmaceutical Sciences
University at Buffalo
The State University of New York
Buffalo, NY, 14214, USA

Y. Xu, Y. Li
Center for Health Research
Guangzhou Institutes of Biomedicine and Health
Chinese Academy of Sciences
Guangzhou 510530, China

Y. Xu, Y. Li
University of China Academyof Sciences
Beijing 100049, China

Y. Tao, M. Li
Guangdong Provincial Key Laboratory of Liver Disease
Guangzhou 510630, China

Experimental Section

Materials

Chloroplatinic acid hexahydrate (H_2PtCl_6), nitro blue tetrazolium chloride (NBT), L-methionine, riboflavin, paraformaldehyde (PFA), and 1,1'-di-n-octadecyl-3,3,3',3'-tetramethylindocarbocyanine perchlorate (DiI) were purchased from Macklin (Shanghai, China). Dimethylamine borane (DMAB), dichloromethane (DCM), polyvinyl alcohol (PVA), nail blue, and carbon tetrachloride (CCl_4) were purchased from Aladdin (Shanghai, China). Bovine serum albumin (BSA) was bought from neoFroxx GmbH (Einhausen, Germany). Cell-culture-grade phosphate buffer solution (PBS) powder was obtained from Biosharp (Beijing, China). Defatted soybean meal was purchased from Yuwang (Yucheng, China). Sodium hyaluronate (SH), neutral protease (NPr), and flaxseed oil were bought from Yuanye (Shanghai, China). Span 80 was purchased from Gersion (Beijing, Chian). Poly (lactic-co-glycolic acid) (PLGA, lactic acid/glycolic acid = 50/50, intrinsic viscosity = 0.70–1.00) was supplied by Polymtek (Shenzhen, China). Horseradish peroxidase (HRP) and Amplex red were obtained from Meilunbio (Dalian, China). Phorbol 12-myristate 13-acetate (PMA) was purchased from Solarbio (Beijing, China). Lipopolysaccharide (LPS) was purchased from Merck (Darmstadt, Germany). Transforming growth factor beta 1 (TGFβ1) was bought from PeproTech (Suzhou, China). H_2O_2 (3%) was purchased from G-Clone (Guangzhou, China). Cell counting kit 8 (CCK8) was purchased from ApexBio (Houston, USA). 4',6-diamidino-2-phenylindole dihydrochloride (DAPI), diethylpyrocarbonate (DEPC)-treated H_2O, and 2',7'-dichlorodihydrofluorescein-diacetate (DCFH-DA) were purchased from Beyotime (Shanghai, China). Tris (4,7-diphenyl-1,10-phenanthroline) ruthenium (II) dichloride complex ($[Ru(dpp)_3]Cl_2$,) was bought from Bidepharm (Shanghai, China). Malonaldehyde (MDA) assay kit, GSH assay kit, and H_2O_2 assay kit were purchased from Boxbio (Beijing, China).

The other reagents in this study were of analytical purity and used without further purification. All the water employed in our investigation was ultrapure, with an electrical resistivity not exceeding 18.2 MΩ cm^{-1}, prepared using a laboratory water purification system (Milli-Q Integral 3, Merck Millipore, Germany).

Methods

Cell Culture

The murine hepatocyte cell line AML12, human monocyte cell line THP1, and human hepatic stellate cell line LX2 were obtained from National Collection of Authenticated Cell Cultures (Shanghai, China). The human hepatocyte cell line LO2 was generously provided as a gift by

the Guangdong Key Laboratory of Liver Disease Research (Guangzhou, China). The murine macrophage cell line Raw264.7 and rat hepatic stellate cell line HSC-T6 were obtained from the American Type Culture Collection. LO2, Raw264.7, LX-2, and HSC-T6 cells were cultured in Dulbecco's modifier Eagle's medium (DMEM) containing 4.5 g L^{-1} glucose (Gibco, USA) supplemented with 10% (volume/volume, v/v) fetal bovine serum (FBS, Biological Industries, Israel) and 1% (w/v) penicillin G sodium/streptomycin sulfate (P/S, Gibco, USA). AML12 cells were cultured in DMEM/F12 (Corning, USA) supplemented with 10% (v/v) FBS, 1% (w/v) P/S, and 10 μg $mL^{–1}$ insulin, 5.5 μg $mL^{–1}$ transferrin, 5 ng $mL^{–1}$ selenium, 40 ng $mL^{–1}$ dexamethasone (Liver Biotechnology, China). THP1 cells were cultured in Roswell Park Memorial Institute-1640 (RPMI-1640) medium (Corning, USA) supplemented with 10% (v/v) FBS, 1% (w/v) PS and 0.05 nM 2-mercaptoethanol (Thermo, USA). All cells were cultured in an incubator (Forma^{TM} Steri-Cycle^{TM} i160, Thermo) at 37 °C under a humidified atmosphere containing 5% CO_2.

Uptake of SecNPs by Cells

Stem cell secretome-encapsulated core-shell nanoparticles (SecNPs) were labeled with DiI by incorporating this dye into the oil phase during the preparation of SecNPs. Subsequently, the pre-cultured cells were co-incubated with DiI-labeled SecNPs for 0, 2, 4, or 6 h. After washing with PBS, the cell nuclei were stained with DAPI, followed by the fixation with 4% (w/v) paraformaldehyde (PFA, pH 7.4) at room temperature for 15 min. After another round of washing, the stained cells were observed and imaged with an inverted fluorescence microscope (Ti2-U, Nikon, Japan), and the DiI^+ fluorescence intensity was determined using a flow cytometer (CytoFLEX S, Beckman, USA).

Effect of SecNPs on Hepatocytes

AML12 or LO2 cells were pre-cultured in a 96-well (5×10^3 cells $well^{–1}$) or 6-well (1×10^6 cells $well^{–1}$) plate for 24 h, followed by serum-starvation for 12 h. Subsequently, the starved cells were treated with CCl_4 (20 mM) for 24 h. After washing with PBS, the treated cells were co-incubated with SecNPs (100, 200, or 300 μg $mL^{–1}$) for 24 h. Finally, the cell viability was evaluated with a CCK-8 assay. The intracellular mRNA level of *Casp3* and *Pcna* (AML12), and *CASP3* and *PCNA* (LO2) were determined using real-time quantitative polymerase chain reaction (RT-qPCR) techniques.

Effect of SecNPs on Macrophages

RAW264.7 cells were pre-cultured in a 6-well plate (1 × 10^6 cells $well^{-1}$) for 24 h, followed by the stimulation with LPS (100 ng mL^{-1}) for an additional 24 h. THP1 monocytes were firstly differentiated into macrophages through the activation with PMA (100 ng mL^{-1}) for 24 h. The matured macrophages were further polarized into pro-inflammatory M1 phenotype with LPS stimulation (100 ng mL^{-1}). After washing with PBS, all M1-polarized macrophages were co-incubated with SecNPs (100, 200, or 300 µg mL^{-1}) for 24 h. The intracellular mRNA levels of *Nos2*, *Tnf*, *Il1b*, *Il10*, *Mrc1*, *Arg1*, and *Tgfb1* (RAW264.7), and *NOS2*, *IL10*, *TGFB1*, and *MMP9* (THP1) were evaluated with RT-qPCR assays.

Effect of SecNPs on HSCs

LX2 or HSC-T6 cells were pre-cultured in a 6-well plate (1 × 10^6 cells $well^{-1}$) for 24 h, followed by the activation with TGFβ1 (10 ng mL^{-1}) for another 24 h. After washing with PBS, the activated hepatic stellate cells (HSCs) were co-incubated with SecNPs (100, 200 or 300 µg mL^{-1}) for 24 h. The intracellular mRNA levels of *ACTA1*, *COL1A1*, *FGF2*, *PDGFB*, *END1*, *TIMP1*, *and TIMP2* (LX2), and *Pdgfa*, *Pdgfb*, *Fgf2*, *Tgfb1*, *Timp1*, *Timp2*, and *Mmp1*3 (HSC-T6) were determined employing RT-qPCR techniques.

Investigation of CAT-Like Activity of PtNZs

The catalase (CAT)-like activity of platinum-based nanozymes (PtNZs) was assessed through sequential H_2O_2 depletion and O_2 generation assays, following the methods outlined in our previous study.[1] Amplex red assay was used to confirm the H_2O_2 consumption by PtNZs. Typically, PtNZs (0, 2, 4, 8 µg mL^{-1}) and H_2O_2 (100 mM) were combined in PBS at 37 °C for 30 min to investigate concentration dependency. PtNZs (4 µg mL^{-1}) and H_2O_2 (100 mM) were mixed in PBS at 37 °C for 0, 15, 30, 45, and 60 min to investigate the time-dependent effects. The reaction was continued for an additional 5 min after the addition and thorough mixing of Amplex red (25 µM) and HRP (7 U mL^{-1}). Then, an ultraviolet-visible (UV-vis) spectral scanning from 450 nm to 650 nm was expeditiously conducted.

The monitoring of O_2 generation resulting from the decomposition of H_2O_2 was further used to assess the CAT-like activity of PtNZs. Specifically, different concentrations of PtNZs (0, 2, 4, or 8 µg mL^{-1}) were mixed with H_2O_2 (50 mM) in 100 mM PBS (15 mL, pH 7.4). The real-time measurement of O_2 generation was conducted using a pen-type dissolved oxygen meter (P6345-01, I-Quip, China).

Investigation of SOD-Like Activity of PtNZs

The superoxide dismutase (SOD)-like activity of PtNZs was assessed using the NBT method, as described in our earlier study.[1] In detail, the riboflavin solution (20 μM), NBT solution (75 μM), methionine solution (13 mM), and PtNZ solution (0, 20, 40, 60, or 80 μg mL^{-1}) were homogeneously mixed in Eppendorf (EP) tubes. PBS was supplemented to ensure equal total volume of solution in each EP tube. After exposure to UV light (254 nm) for 5 min, a rapid UV-Vis spectral scanning from 300 nm to 800 nm was performed, with peak absorption observed at 560 nm. The SOD-like activity of PtNZs is inversely proportional to the intensity of blue color of the reaction solution.

Intracellular Antioxidant Efficiency of PtNZs

The cell-permeable DCFH-DA was applied to indicate the ROS levels in hepatocytes. AML12 or LO2 cells were first cultured in a 24-well plate (2×10^4 cells $well^{-1}$) for 24 h, followed by the co-incubation with PtNZs (0, 2, 4, or 8 μg mL^{-1}) for 12 h. Subsequently, all cells were exposed to H_2O_2 (500 μM) for an additional 12 h. After washing with PBS, the treated AML12 or LO2 cells were stained with DCFH-DA at 37 °C for 30 min. Following an additional round of washing, the fluorescence images were captured, and the flow cytometry analysis was performed to quantify the results.

Intracellular O_2 Generation of PtNZs

$[Ru(dpp)_3]Cl_2$, an O_2-indicated fluorescence probe, was used to determine the O_2 generation level in HSCs. LX2 or HSC-T6 cells were initially seeded in a 12-well plate (5×10^4 cells $well^{-1}$) and cultured for 12 h. Then, all cells were transferred into a hypoxic environment (1% O_2) created in a small tris-gas incubator (Galaxy 48 R, Eppendorf, Germany), followed by another 12 h of culture. After that, the cells were co-incubated with PtNZs (0, 2, 4, or 8 μg mL^{-1}) under hypoxic conditions for 12 h. The uninternalized PtNZs were removed by washing. Subsequently, the washed cells were concurrently co-incubarted with H_2O_2 (500 μM) and $[Ru(dpp)_3]Cl_2$ in hypoxic environment for 4 h. Lastly, the fluorescence images were captured, and the flow cytometry analysis was performed.

Extraction and Isolation of SPI

Soy protein isolate (SPI) was extracted and isolated according to the methods described in our previous report.[2] Briefly, the defatted soy meal underwent further grinding, defatting, and drying processes before being completely dispersed in ultrapure water. The initial pH of the

dispersion was adjusted to 8.0 using a NaOH solution, followed by continuous agitation for 2 h, during which the pH was readjusted to 8.0 every 30 min using a 1 M NaOH solution. The pH of supernatant obtained by centrifugation at 8 000 g was adjusted to 4.5 by adding a HCl solution. Then, the precipitate was gathered via centrifugation at 5 000 g, followed by redispersion in ultrapure water. A clear crude protein solution was obtained after gradually adjusting the pH to 7.0, and subsequently transferred into dialysis tubes with a molecular weight cut-off range of 8–14 kDa. Following thorough dialysis in vigorously stirred ultrapure water, the resulting dialysate was collected and subjected to lyophilization to obtain SPI powder.

Effect of SPI on Hepatocytes and Injured Hepatocytes

LO2 cells were initially cultured in a 96-well plate (5×10^3 cells $well^{-1}$) for 24 h. To simulate hepatocyte injury, the cells were treated with 500 μM H_2O_2 for 12 h. After washing with PBS, the normal or injured LO2 cells were co-incubated with 0.75% (w/v) SPI or NPr-hydrolyzed SPI for 24 h. Finally, the cell viability was assessed using a CCK-8 assay.

Responsive Degradation and Drug Release of MN Arrays

To evaluate the *in vitro* release property and predict the *in vivo* drug release behavior of microneedle (MN) arrays, Nile blue (NB) was encapsulated into MNs as a drug surrogate. The NB-loaded MNs were immersed into PBS (pH 7.4) at 37 °C under dark conditions. At predetermined time intervals, 100 μL of release medium (PBS) was collected, and an equal volume of fresh PBS was supplemented. After 1 h of release monitoring, a subset of the samples were subjected to heating at 45 °C for 5 min in a water bath to simulate the *in vivo* photothermal effect induced by NIR irradiation. The degradation of MN arrays and the release of drugs were continuously monitored during the experiment by quantifying the fluorescence intensity (excited at 630 nm) of the release medium using a fluorescence spectrophotometer (RF6000, Shimadzu, Japan). After obtaining a standard curve correlating the concentration of NB in PBS with fluorescence intensity, the calculation of cumulative release rate was performed.

Transcriptome Analysis of Liver Tissues

Transcriptome analysis was performed on liver tissues from the control and PSMN+NIR (treatment through PtNZ+SecNP-loaded MN patch implantation and NIR irradiation) groups. The RNA extraction, purification, fragmentation, reverse transcription, PCR amplification, library construction, and sequencing procedures were conducted by LC-Bio Technology

(Hangzhou, China). Subsequently, the acquired data underwent meticulous analysis and visualization following the methodologies outlined in our and others' previous studies.[1a, 2-3]

Histologic Evaluation

The sampled liver tissues were fixed in 4% (w/v, pH 7.4) PFA solutions at room temperature for at least 1 day. Subsequently, the fixed samples were dehydrated using gradient ethanol solutions and finally embedded in paraffin. The embedded tissues were sectioned into slices with a constant thickness of 6 μm employing a paraffin microtome (RM 2235, Leica, Germany). Following thermal melting at 60 °C, deparaffination with xylene, and subsequent rehydration using counter-gradient ethanol solutions, the liver sections underwent staining procedures employing hematoxylin and eosin (H&E), Sirius red, Masson's trichrome, and terminal deoxynucleoitidyl transferase-mediated deoxyuridine triphosphate nick end labeling (TUNEL) assays.

For immunofluorescence staining, the rehydrated liver tissue sections were subjected to antigen retrieval, and subsequently treated with a blocking solution containing 5% (v/v in PBS) normal goat serum prior to immunofluorescence staining. After washing with PBS, the tissue sections were separately co-incubated overnight at 4 °C with rabbit-derived primary antibodies against αSMA (GB111364, Servicebio, China), Col1a1 (GB114197, Servicebio), iNOS (GB11119, Servicebio), Arg1 (GB11285, Servicebio), and Ki67 (GB111499, Servicebio). Subsequently, the liver tissue sections were thoroughly washed and co-incubated with goat-derived anti-rabbit secondary antibodies conjugated with Alexa Fluor 488 (GB25303, Servicebio) or Cyanine 3 (GB21303, Servicebio) in the dark. Following another round of washing, the tissue sections were mounted using an antifade medium containing DAPI. Finally, bright-field and fluorescence images of stained tissue sections were visualized and captured utilizing an inverted fluorescence microscope (Ti2-U, Nikon) and analyzed quantitatively using Fiji software (version 1.53c, USA).

Biosafety Evaluation

To evaluate the safety of our implemented treatments, we utilized an automated biochemical analyzer (3100, Hitachi, Japan) to conduct serological testing on serum samples obtained by centrifuging blood at 4 °C for 20 min at a speed of 1 000 g. Additionally, histological analyses of harvested organs were performed using H&E staining and microscopic examination.

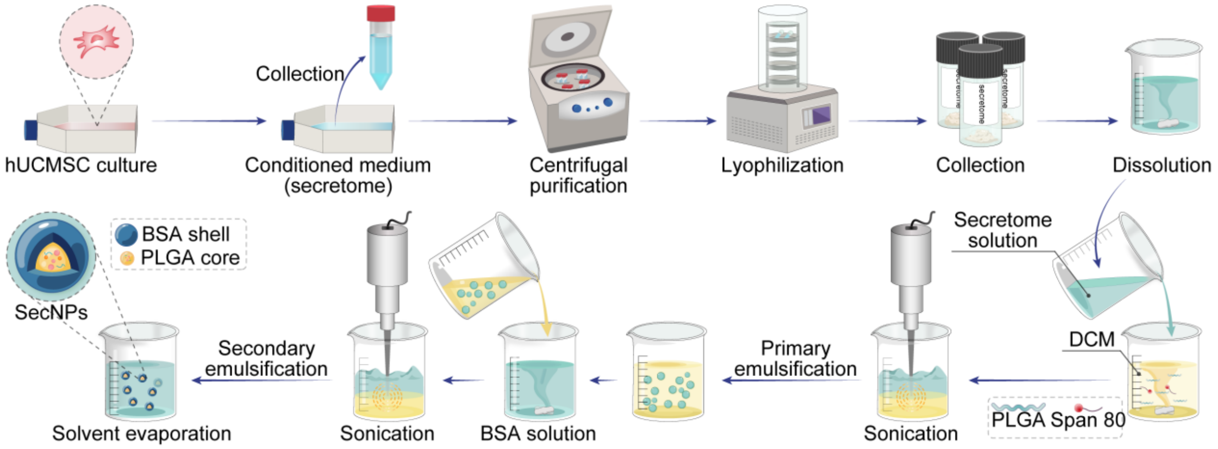

Figure S1. Fabrication of SecNPs. The conditioned medium of hUCMSCs, devoid of serum, was collected and subjected to centrifugation, filtration, and lyophilization to obtain the secretome powder. Subsequently, the secretome was redissolved and encapsulated within the PLGA core of SecNPs employing double emulsification and solvent evaporation techniques.

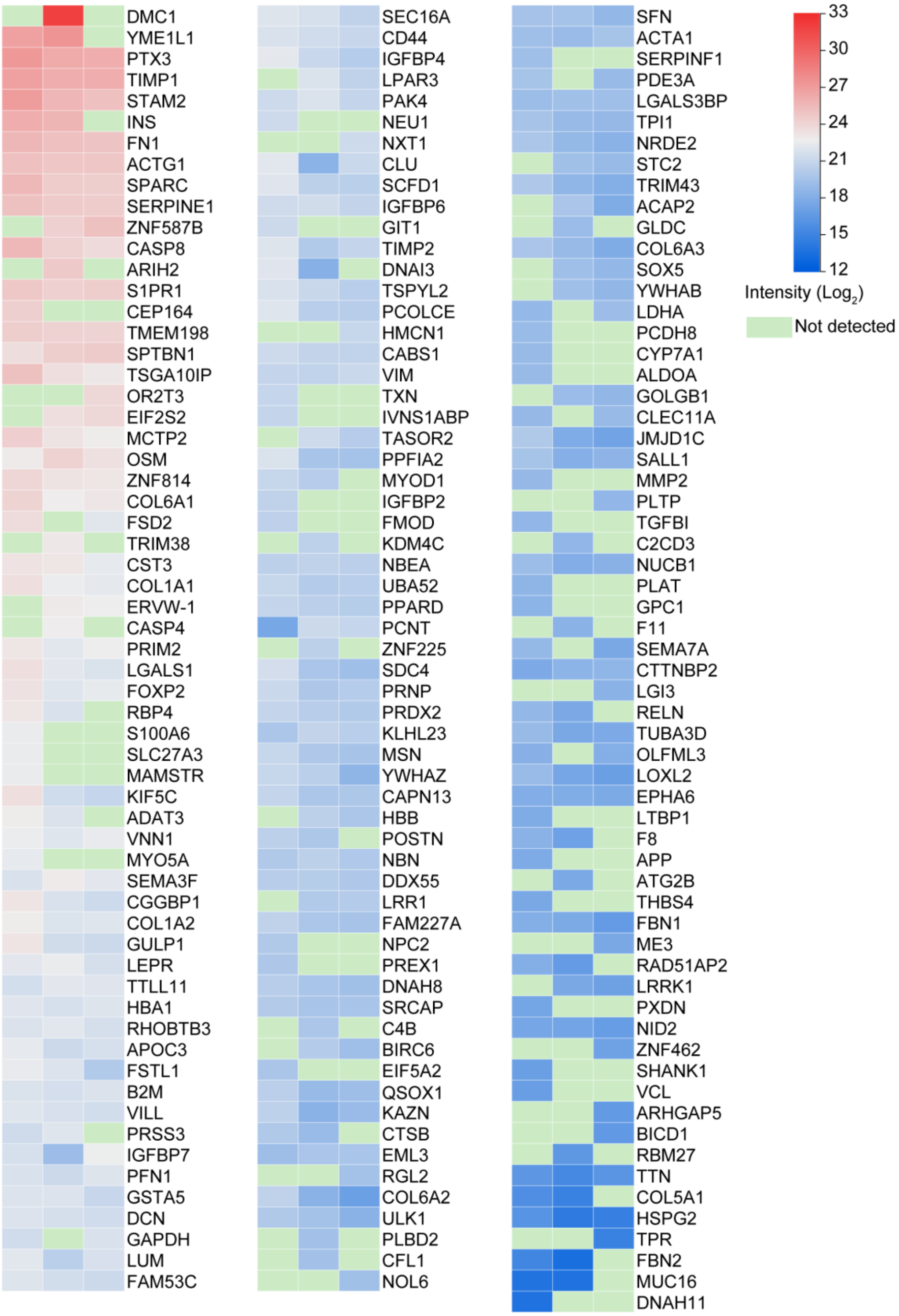

Figure S2. Heatmap illustrating all proteins and their intensities in hUCMSC secretome, analyzed using label-free quantitative methods.

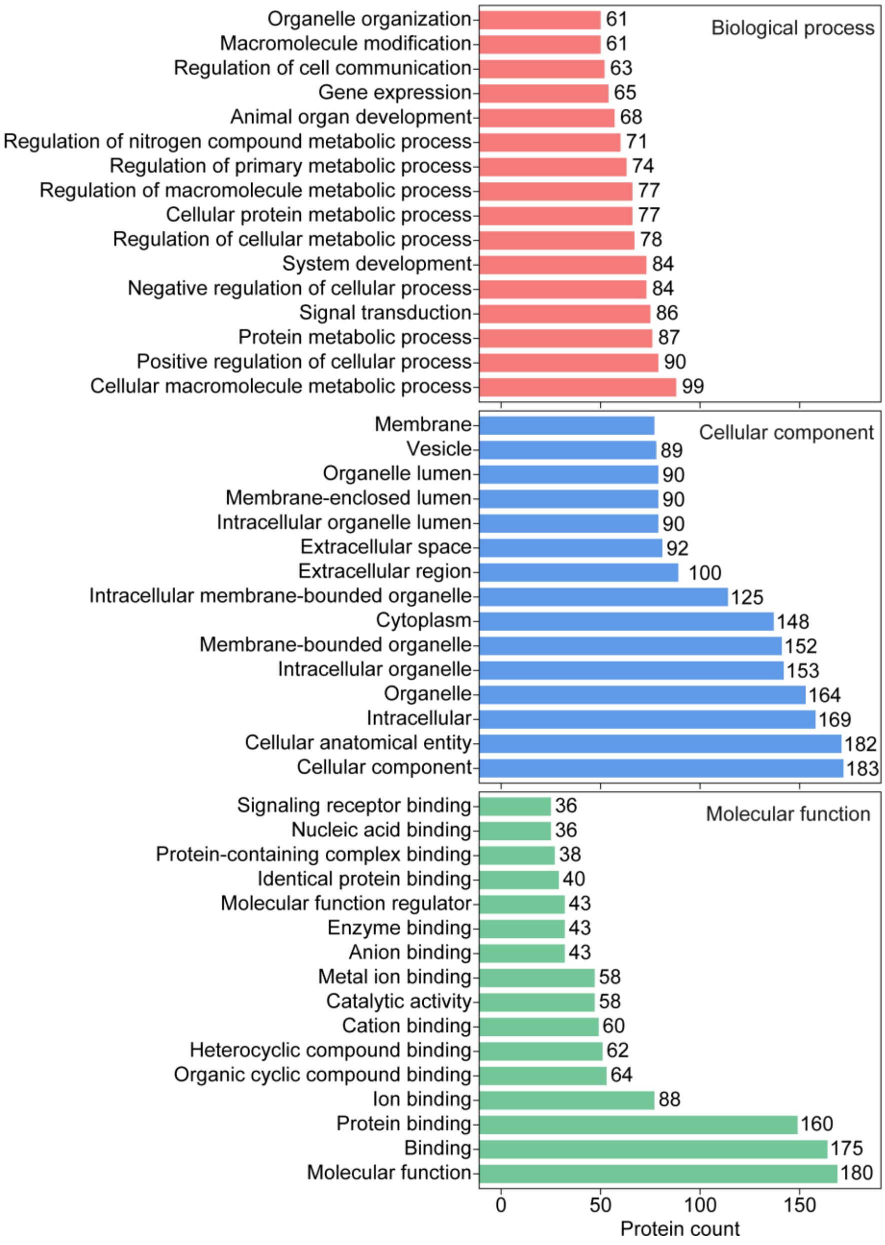

Figure S3. Enrichment analysis of gene ontology (GO) terms associated with all proteins in hUCMSC secretome, providing insights into their biological processes, cellular components, and molecular functions.

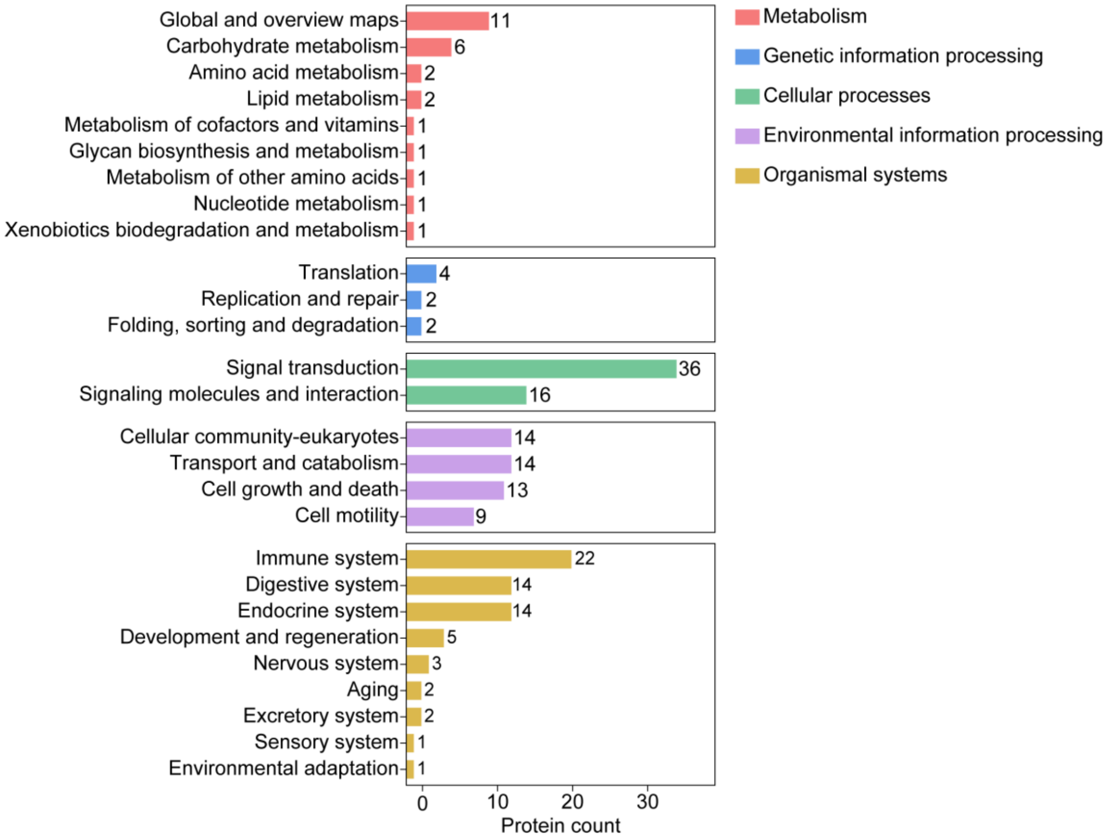

Figure S4. Enrichment analysis performed on Kyoto encyclopedia of genes and genomes (KEGG) pathways of all proteins in hUCMSC secretome.

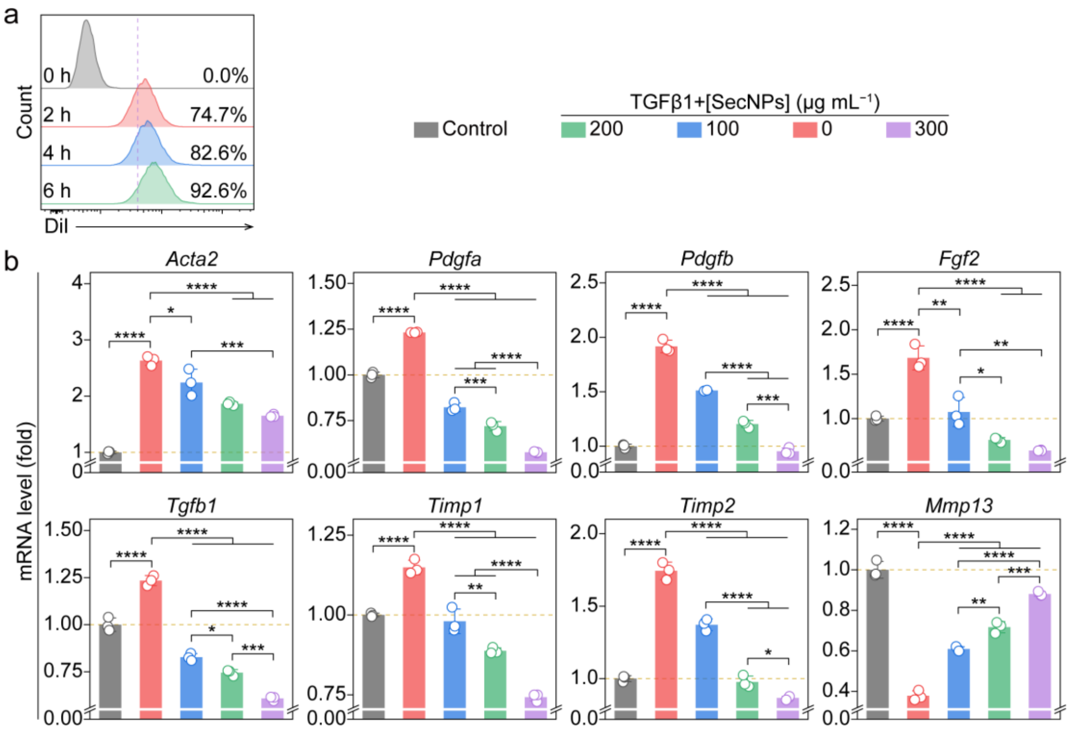

Figure S5. SecNP uptake by HSCs and its phenotype-regulating effect. a) Representative flow cytometry histograms illustrating the internalization of DiI-labeled SecNPs by HSC-T6 cells during various co-incubation time periods. b) Relative intracellular levels of mRNA associated with ECM deposition (*Aata2*), HSC activation (*Pdgfa*, *Pdgfb*, *Fgf2*, and *Tgfb1*), and ECM degradation (*Timp1*, *Timp2*, and *Mmp13*) evaluated using HSC-T6 cells. Data are presented as mean ± SD (n = 3). Statistical significances were assessed using one-way ANOVA followed by Tukey's multiple comparisons post hoc test. *, $P < 0.05$; **, $P < 0.01$; ***, $P < 0.001$; and ****, $P < 0.0001$.

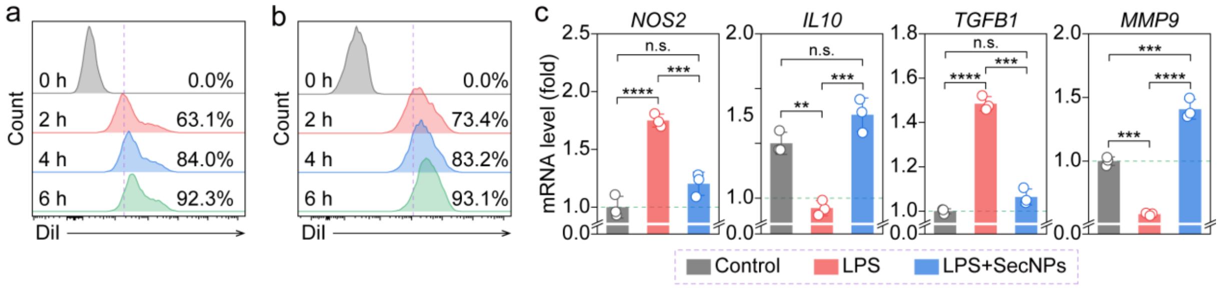

Figure S6. SecNP uptake by macrophages and its phenotype-regulating effect. a, b) Representative flow cytometry histograms depicting the internalization of DiI-labeled SecNPs by RAW264.7 (a) and THP1 (b) cells over various various co-incubation periods. c) Relative intracellular levels of mRNA involved in proinflammation *(NOS2*), anti-inflammation (*IL10*), HSC activation (*TGFB1*), or ECM degradation (*MMP9*) pathways in THP1 cells subjected to the indicated treatments. Data are shown as mean ± SD (n = 3). Statistical significances were evaluated with one-way ANOVA followed by Tukey's multiple comparisons post hoc test. **, $P < 0.01$; ***, $P < 0.001$; ****, $P < 0.0001$; and n.s., not significant.

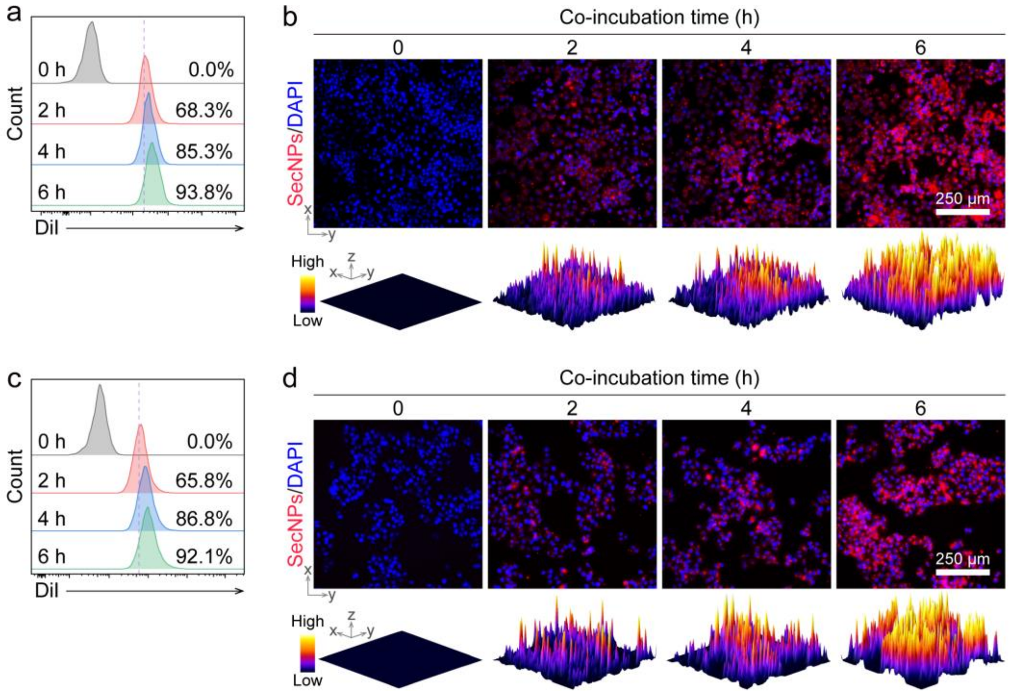

Figure S7. Uptake of SecNPs by hepatocytes. a–d) Internalization of DiI-labeled SecNPs by AML12 (a, b) and LO2 (c, d) cells illustrated by representative flow cytometry histograms (a, c) and representative fluorescence images with corresponding quantified 3D surface plots (b, d) over various co-incubation time periods.

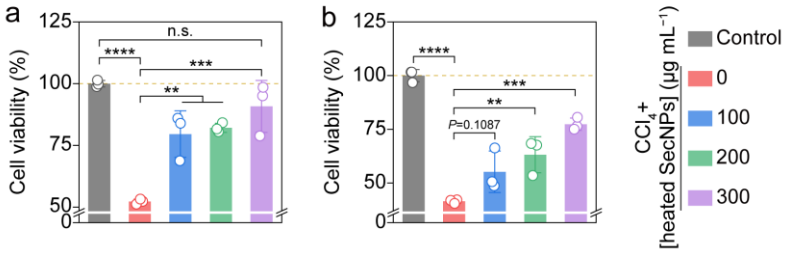

Figure S8. Influence of pre-heating on the protective effect of SecNPs against the CCl_4-induced hepatocyte damage *in vitro*. a, b) Cell viabilities of AML12 (a) and LO2 (b) cells, which were exposed to CCl_4 and subsequently treated with SecNPs after being pre-heated at 45 °C for 30 min. Data are presented as mean ± SD (n = 3). Statistical significances were estimated employing one-way ANOVA followed by Tukey's multiple comparisons post hoc test. **, $P < 0.01$; ***, $P < 0.001$; ****, $P < 0.0001$; and n.s., not significant.

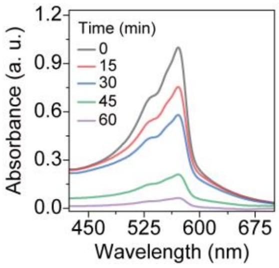

Figure S9. CAT-like activity of PtNZs evidenced by the of H_2O_2 consumption over various catalysis time periods.

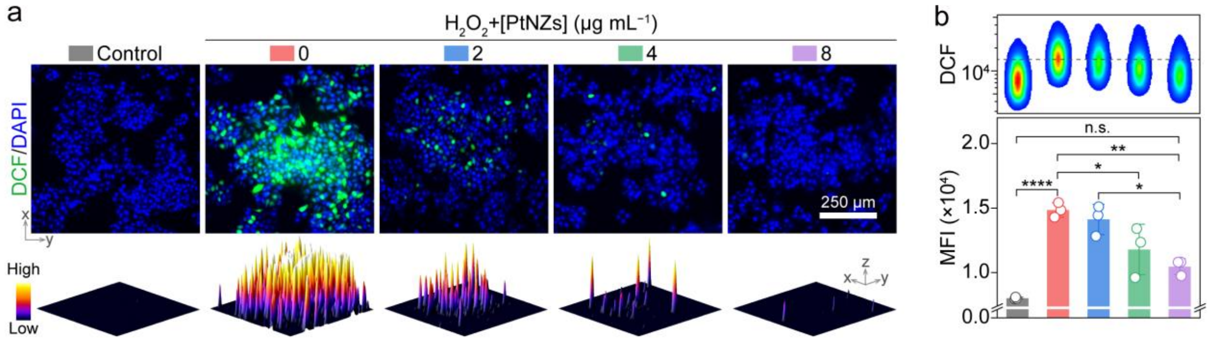

Figure S10. Intracellular ROS-depleting activity of PtNZs. a) Representative DCF^+ fluorescence images and corresponding 3D surface plots quantifying ROS levels in LO2 cells treated as indicated. b) Representative DCF^+ flow cytometry pseudo-color plots and corresponding quantifications in LO2 cells following various treatments. Data are presented as mean ± SD (n = 3). Statistical significances were assessed using one-way ANOVA with Tukey's multiple comparisons post hoc test. *, $P < 0.05$; **, $P < 0.01$; ****, $P < 0.0001$; and n.s., not significant.

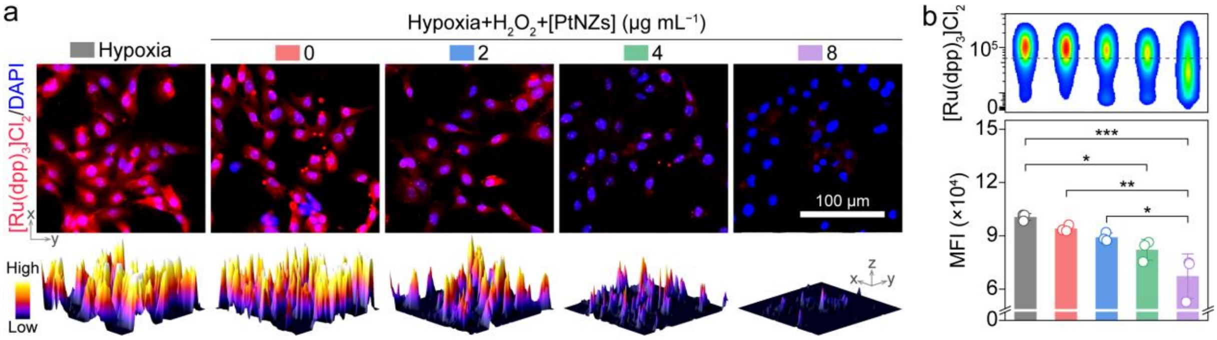

Figure S11. Intracellular catalytic activity of PtNZs in converting H_2O_2 to O_2. a) Representative fluorescence images and quantified 3D surface plots using $[Ru(dpp)_3]Cl_2^+$ as a hypoxia indicator, demonstrating the ability of PtNZs to alter intracellular hypoxia levels in HSC-T6 cells under various treatments. b) Representative $[Ru(dpp)_3]Cl_2^+$ flow cytometry pseudo-color plots and corresponding quantifications in HSC-T6 cells subjected to the indicated treatments. Data are shown as mean ± SD (n = 3). Statistical significances were estimated using one-way ANOVA followed by Tukey's multiple comparisons post hoc test. *, $P < 0.05$; **, $P < 0.01$; and ***, $P < 0.001$.

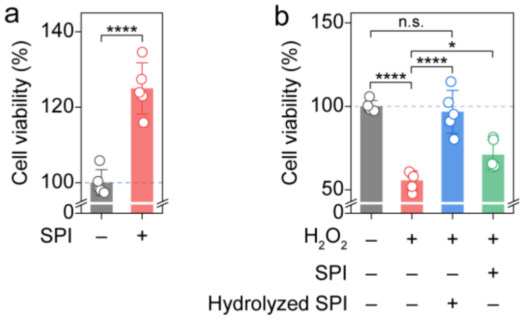

Figure S12. *In vitro* protective effect of SPI in the thermal-responsive MNs. a) Cell viabilities of LO2 cells following the co-incubation with or without native SPI. b) Cell viabilities of LO2 cells sequentially undergoing the treatment with H_2O_2, and native SPI or NPr-hydrolyzed SPI. Data are shown as mean ± SD (n = 5). Statistical significances were evaluated by one-way ANOVA with Tukey's multiple comparisons post hoc test. *, $P < 0.05$; ****, $P < 0.0001$; and n.s., not significant.

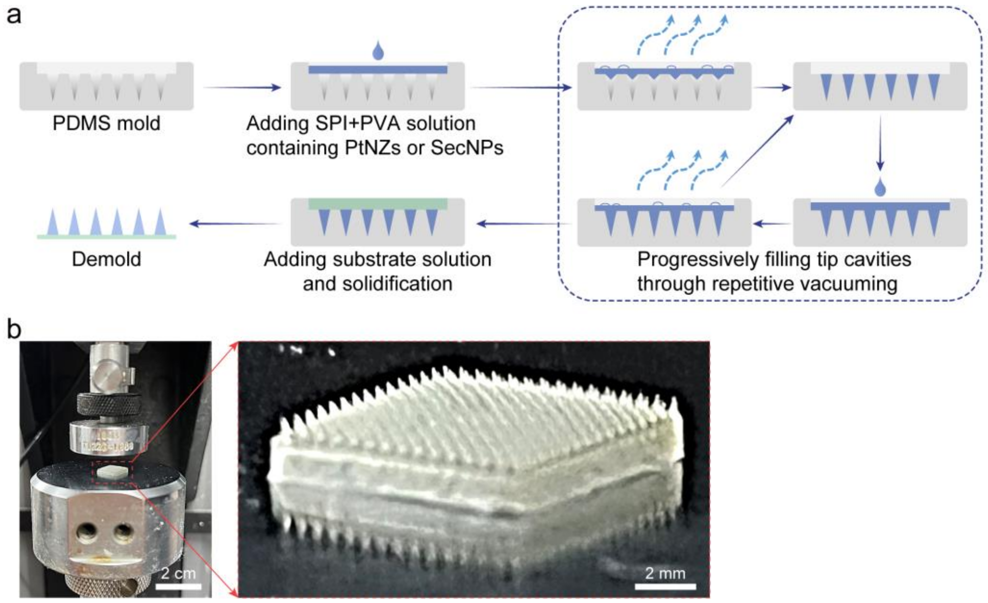

Figure S13. a) Schematic illustration of fabricating MN arrays. b) Representative visual appearance of a microneedle patch undergoing the mechanical compression test.

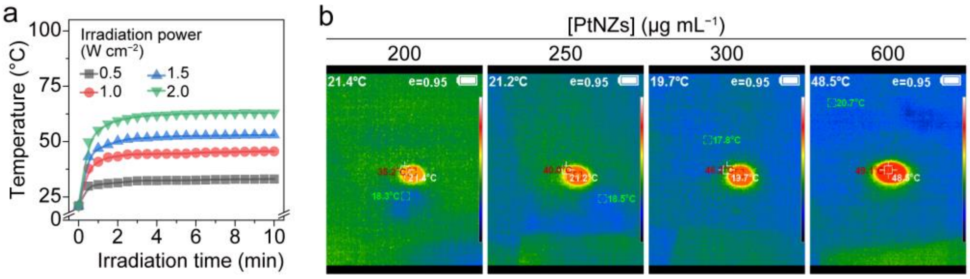

Figure S14. Photothermal effect of PMNs. a) Photothermal temperature curves of PMN arrays under NIR irradiation at varying power levels. b) Representative infrared thermal images of PMN arrays containing different concentrations of PtNZs.

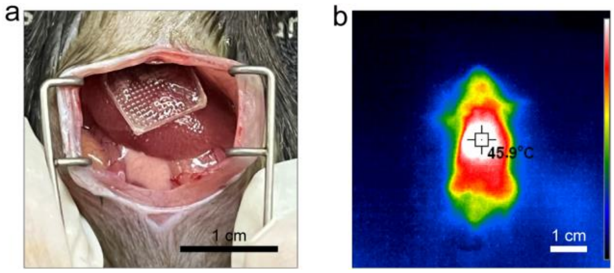

Figure S15. Surgical implantation and treatment with MN arrays. a) Representative digital image capturing the process of laparotomy and implantation of an MN patch in a mouse. b) Representative infrared thermal image of the mouse post-implantation of the MN arrays containing PtNZs during exposure to NIR irradiation, indicating the *in situ* photothermal effects.

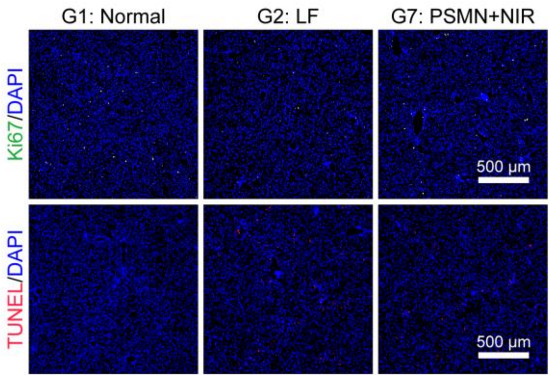

Figure S16. *In vivo* analysis of proliferation and apoptosis in liver tissues. Representative Ki67^{+} (indicating cellular proliferation) immunofluorescence and TNUEL^{+} (reflecting apoptosis) fluorescence images of liver tissues from the normal, liver fibrosis (LF), and PSMN+NIR groups.

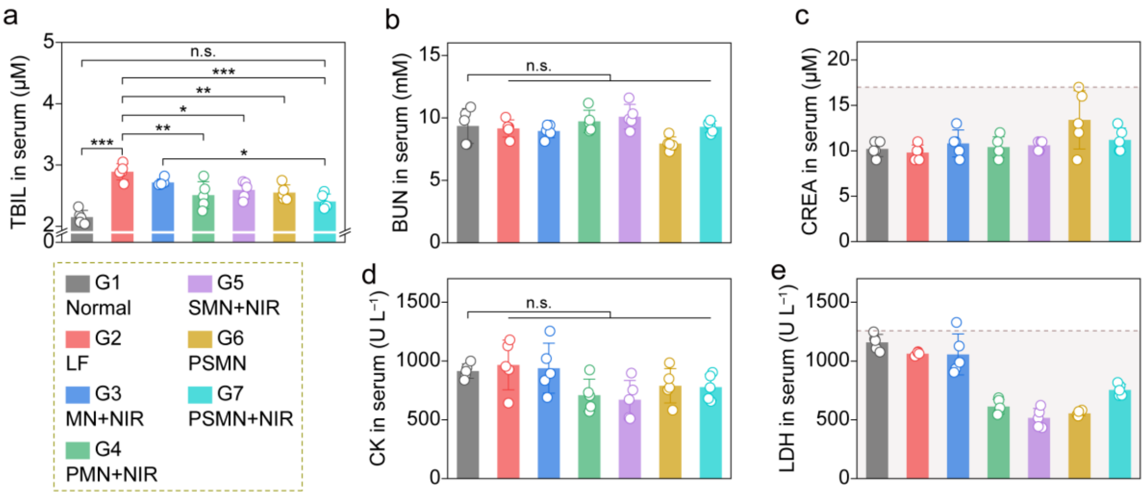

Figure S17. Assessment of liver function recovery and biosafety based on blood biochemical indices. a–e) Biochemical index levels of TBIL (liver function) (a), BUN (kidney function) (b), CREA (kidney function) (c), CK (heart function) (d), and LDH (heart function) (e) in the serum from the mice receiving indicated treatments. Dash lines in panels c and e indicate the recommended maximum levels of serum biochemical indices.[2] Data are shown as mean ± SD (n = 5). Statistical significances were assessed using one-way ANOVA followed by Tukey's multiple comparisons post hoc test. *, $P < 0.05$; **, $P < 0.01$; ***, $P < 0.001$; and n.s., not significant. G1, normal group; G2, liver fibrosis (LF) model group without treatment; G3, treatment through blank MN patch implantation and NIR irradiation (MN+NIR); G4, treatment through PtNZ-loaded MN (PMN) patch implantation and NIR irradiation (PMN+NIR); G5, treatment through SecNP-loaded MN (SMN) patch implantation and NIR irradiation (SMN+NIR); G6, treatment through PtNZ+SecNP-loaded MN (PSMN) patch implantation (PSMN); G7: treatment through PSMN patch implantation and NIR irradiation (PSMN+NIR).

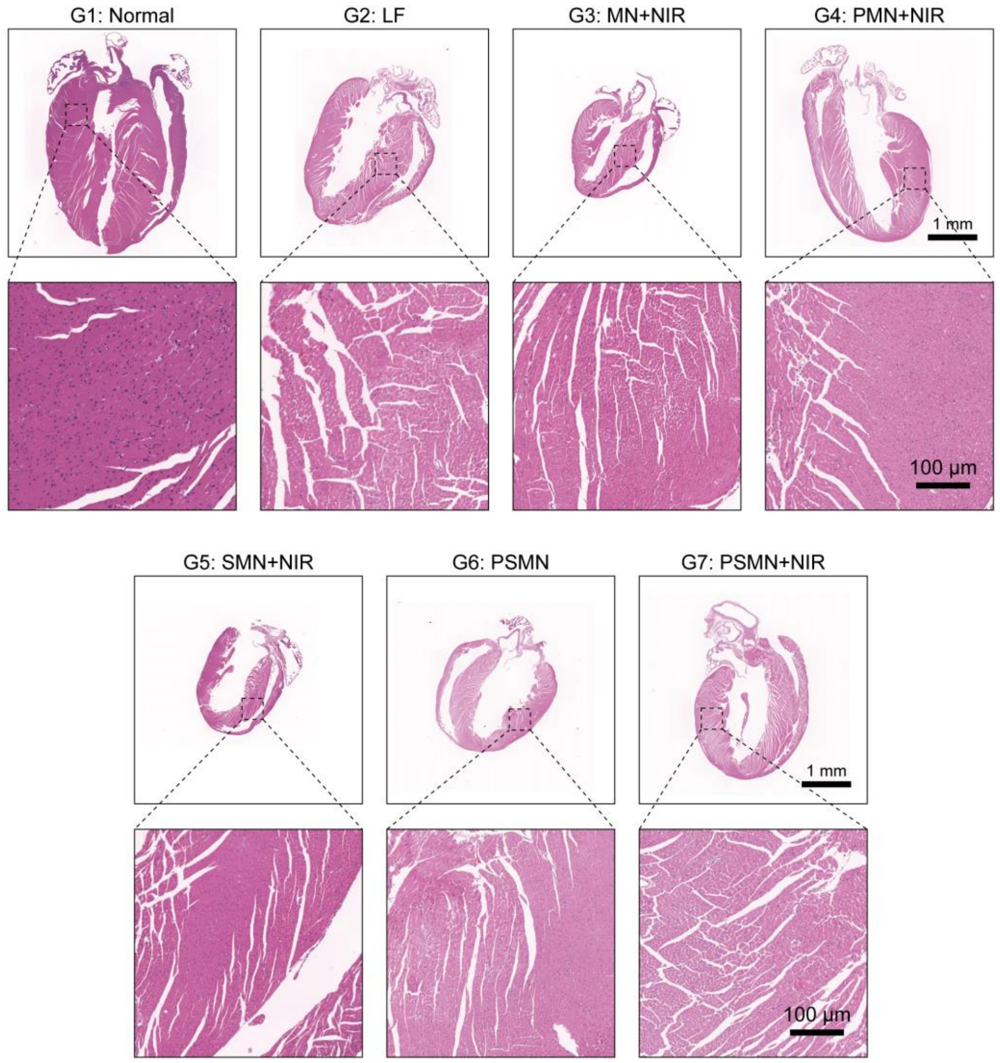

Figure S18. Biosafety determined through the histological evaluation. Representative H&E-stained images of hearts from mice in the indicated groups.

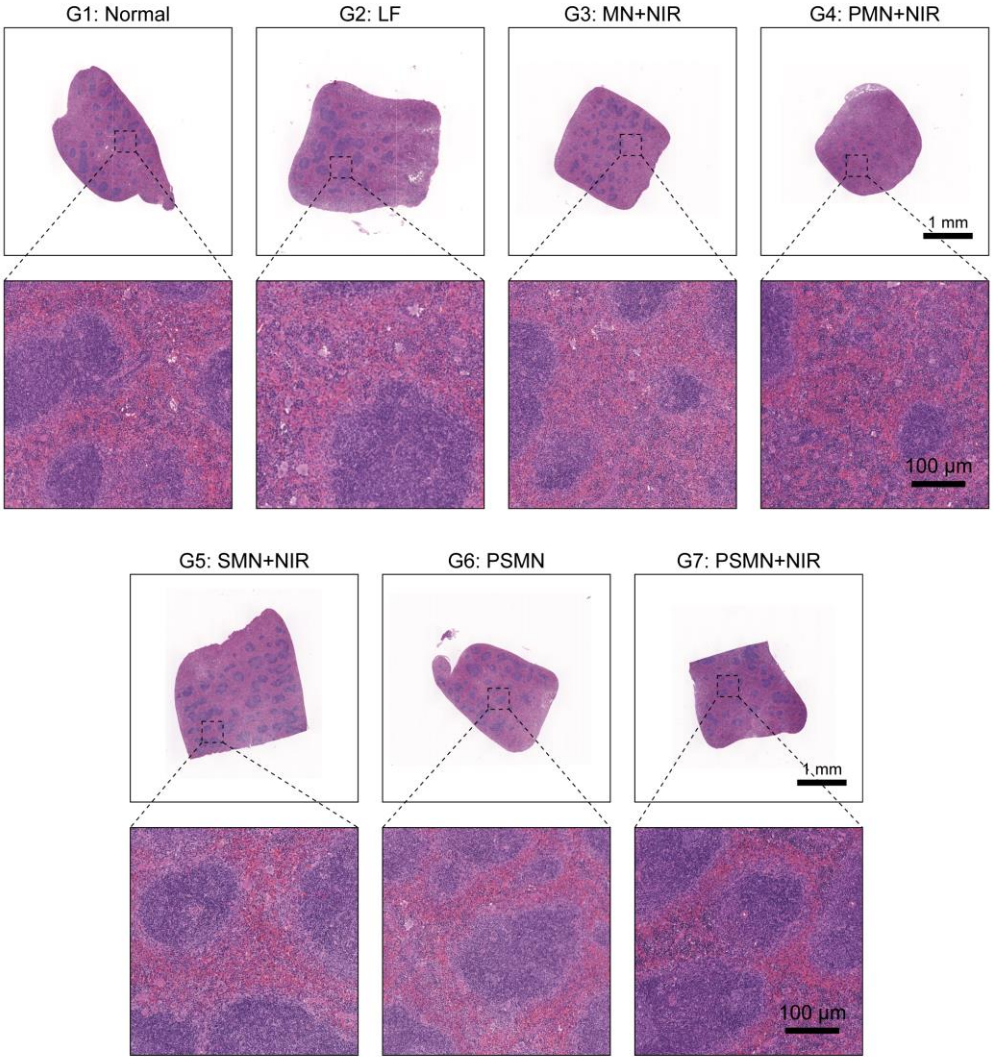

Figure S19. Biosafety determined through the histological evaluation. Representative H&E-stained images of spleens from mice in the indicated groups.

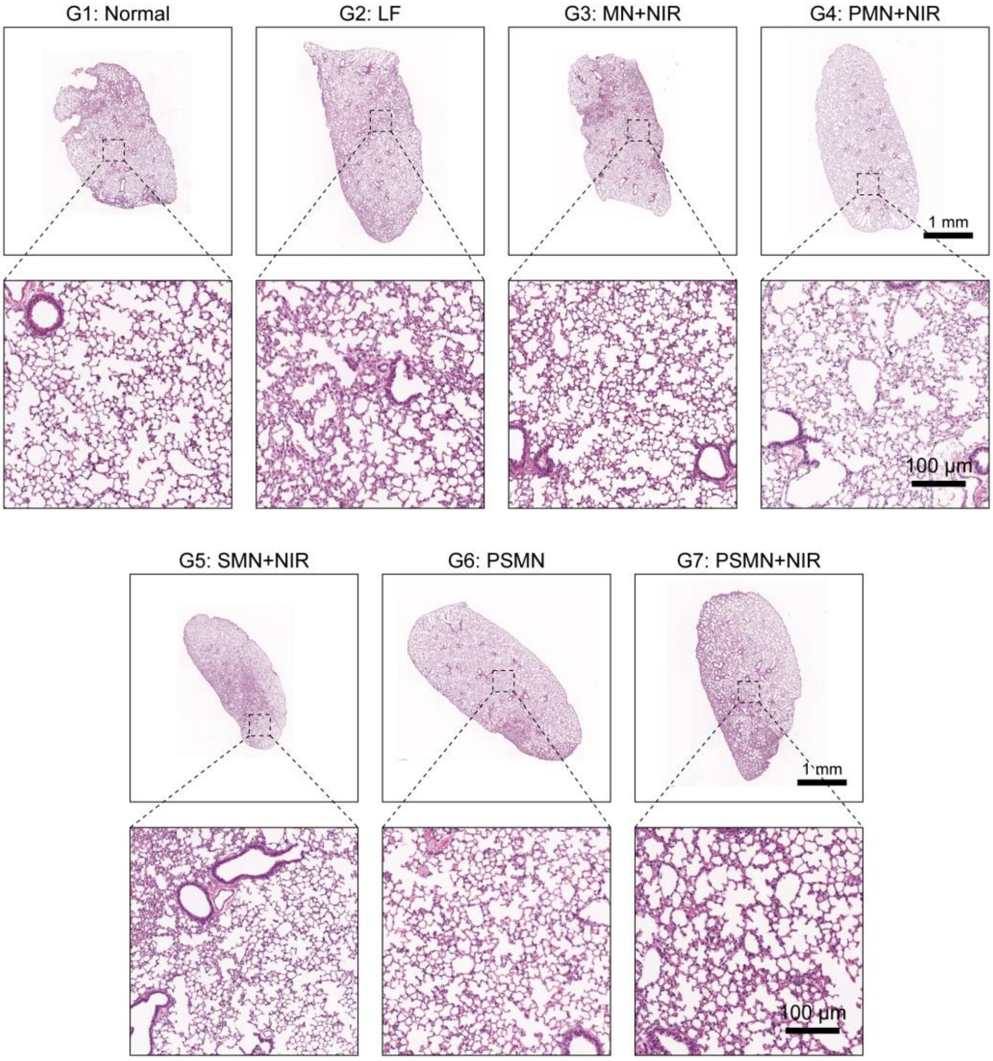

Figure S20. Biosafety determined through the histological evaluation. Representative H&E-stained images of lungs from mice undergoing the designated treatments.

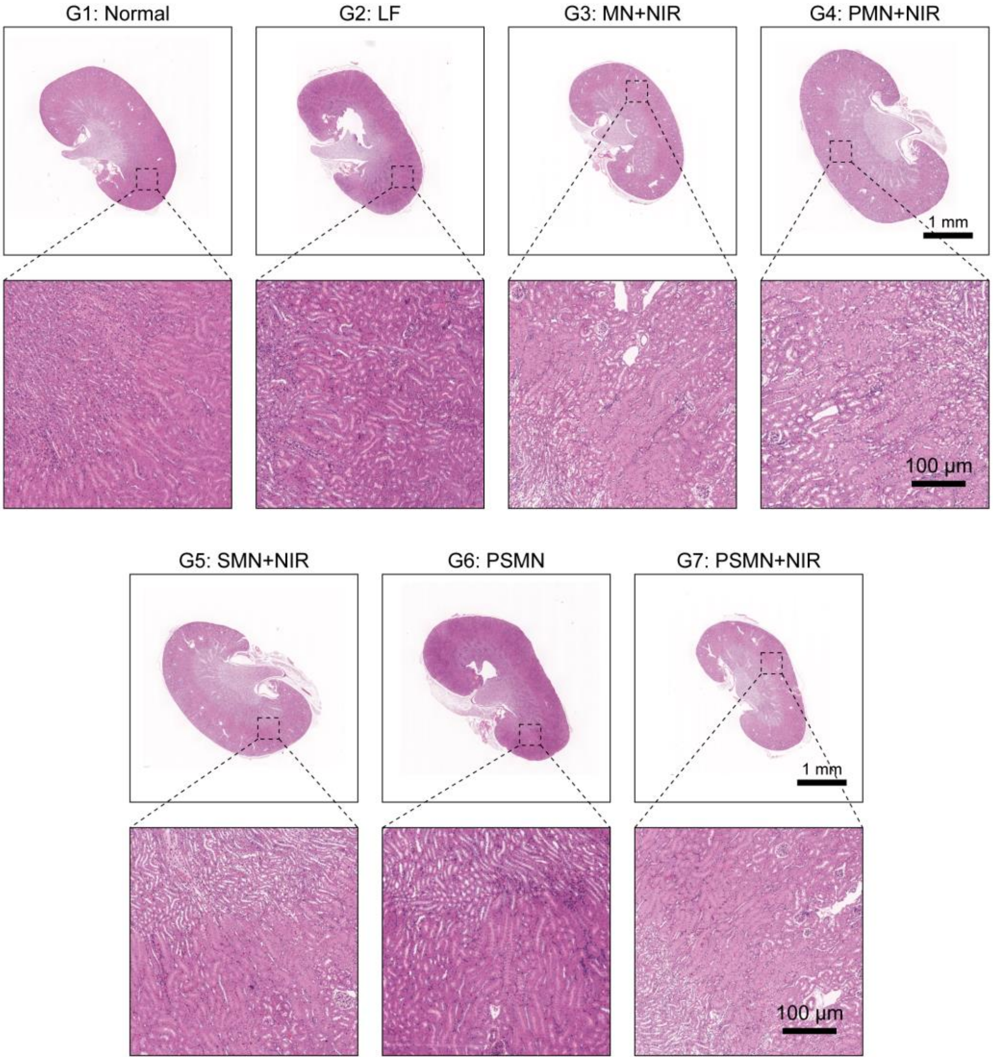

Figure S21. Biosafety determined through the histological evaluation. Representative H&E-stained images of kidneys from mice in the designated groups.

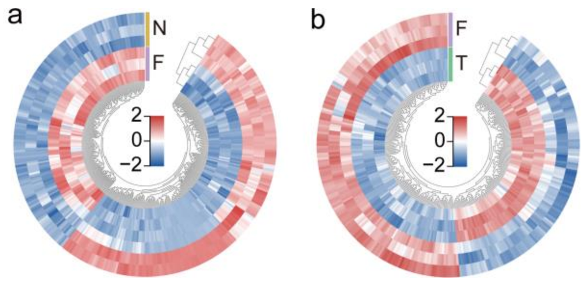

Figure S22. Interactive heatmap and clustering analysis of differentially expressed genes (DEGs) (P < 0.05 and FC > 2 or FC < 0.5). a) Normal group (N) versus LF group (F). b) LF group (F) versus PSMN+NIR group (treatment, T).

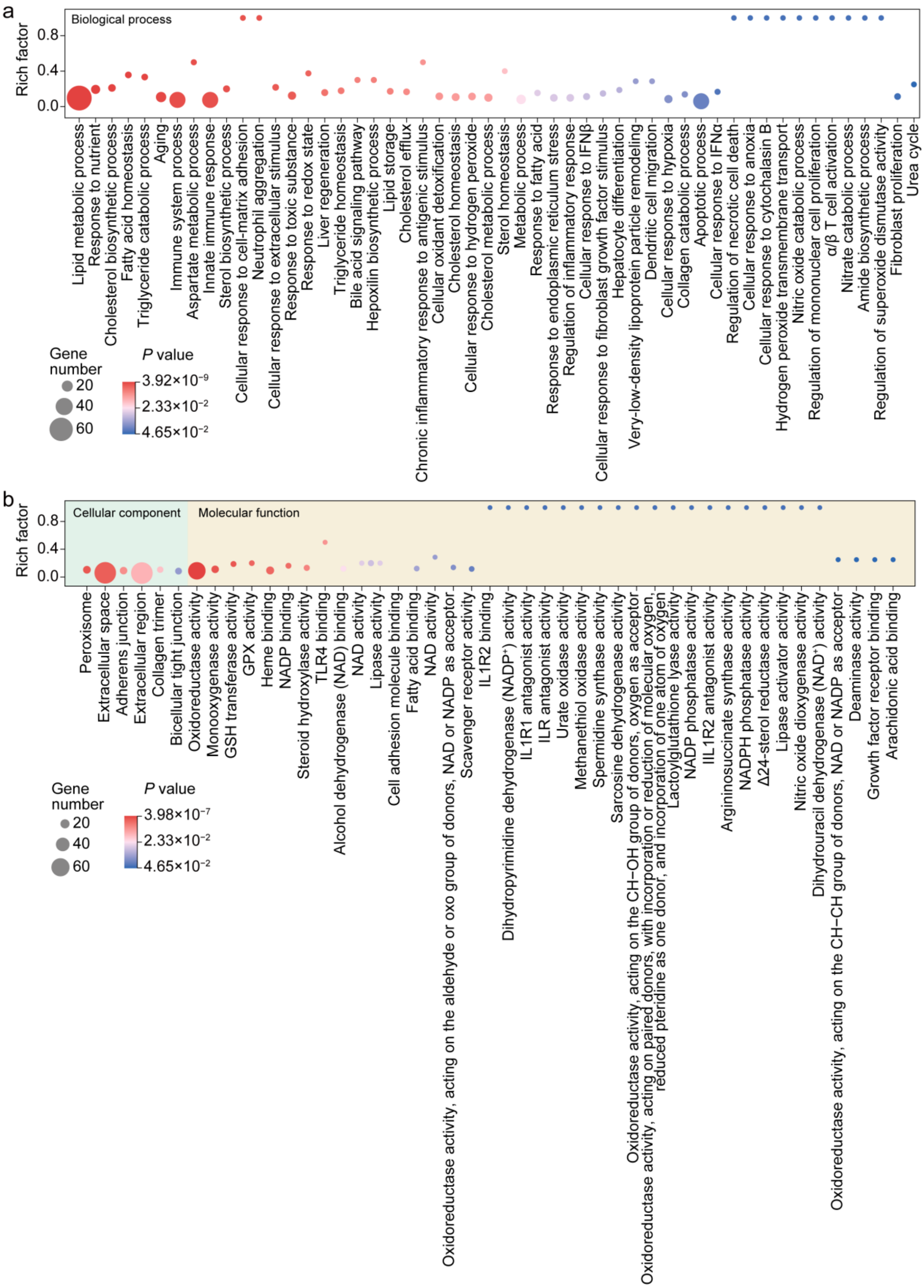

Figure S23. Enrichment analysis performed on GO terms of DEGs in the liver tissues from LF group and normal group. a) GO terms associated with biological process. b) GO terms implicated in cellular component and molecular function.

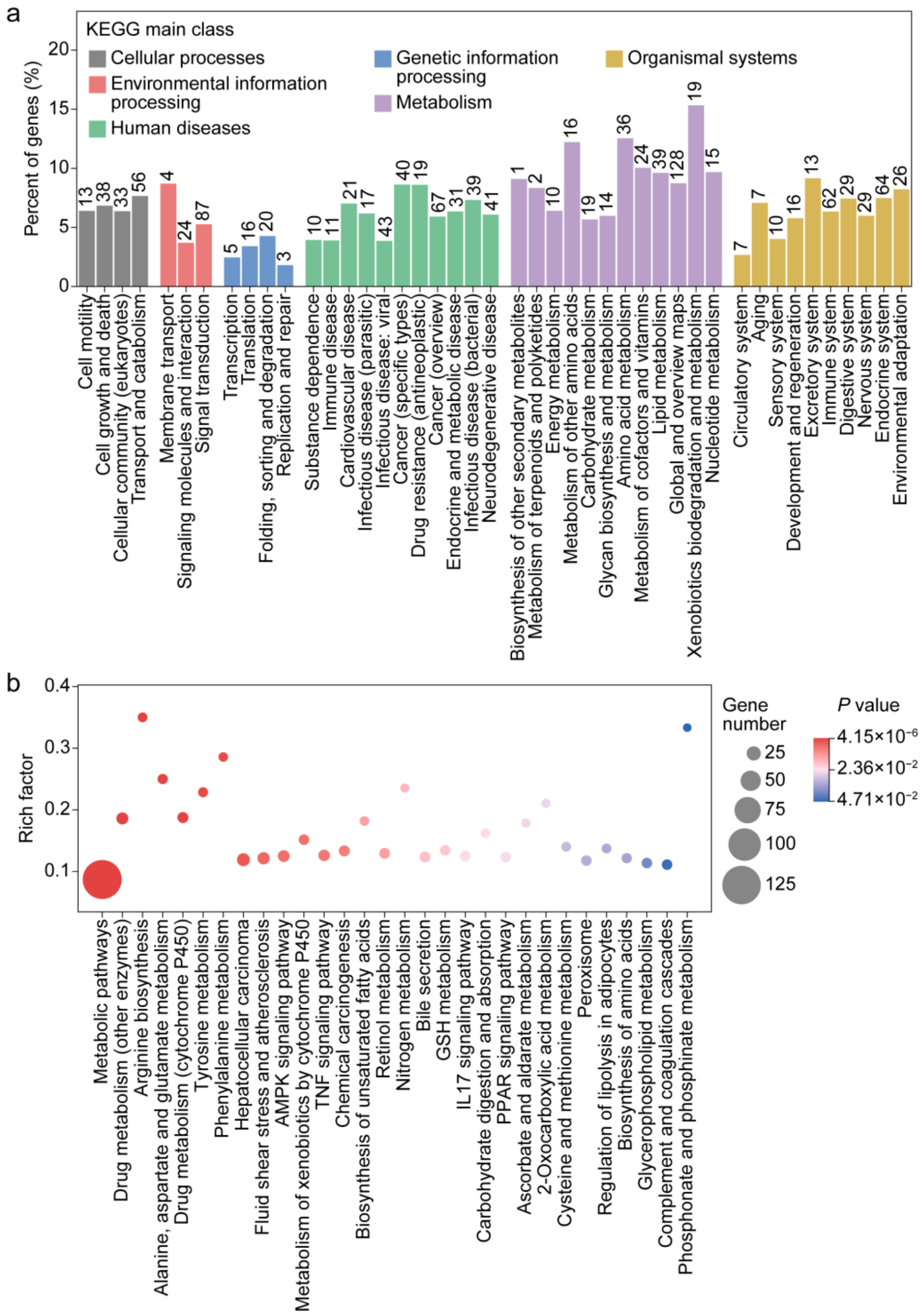

Figure S24. Enrichment analysis performed on KEGG main classes and pathways of DEGs in the liver tissues from LF group and normal group. a) Percent of DEGs enriched in various pathways assigned to different KEGG main classes. b) Rich factor of DEGs in different KEGG pathways.

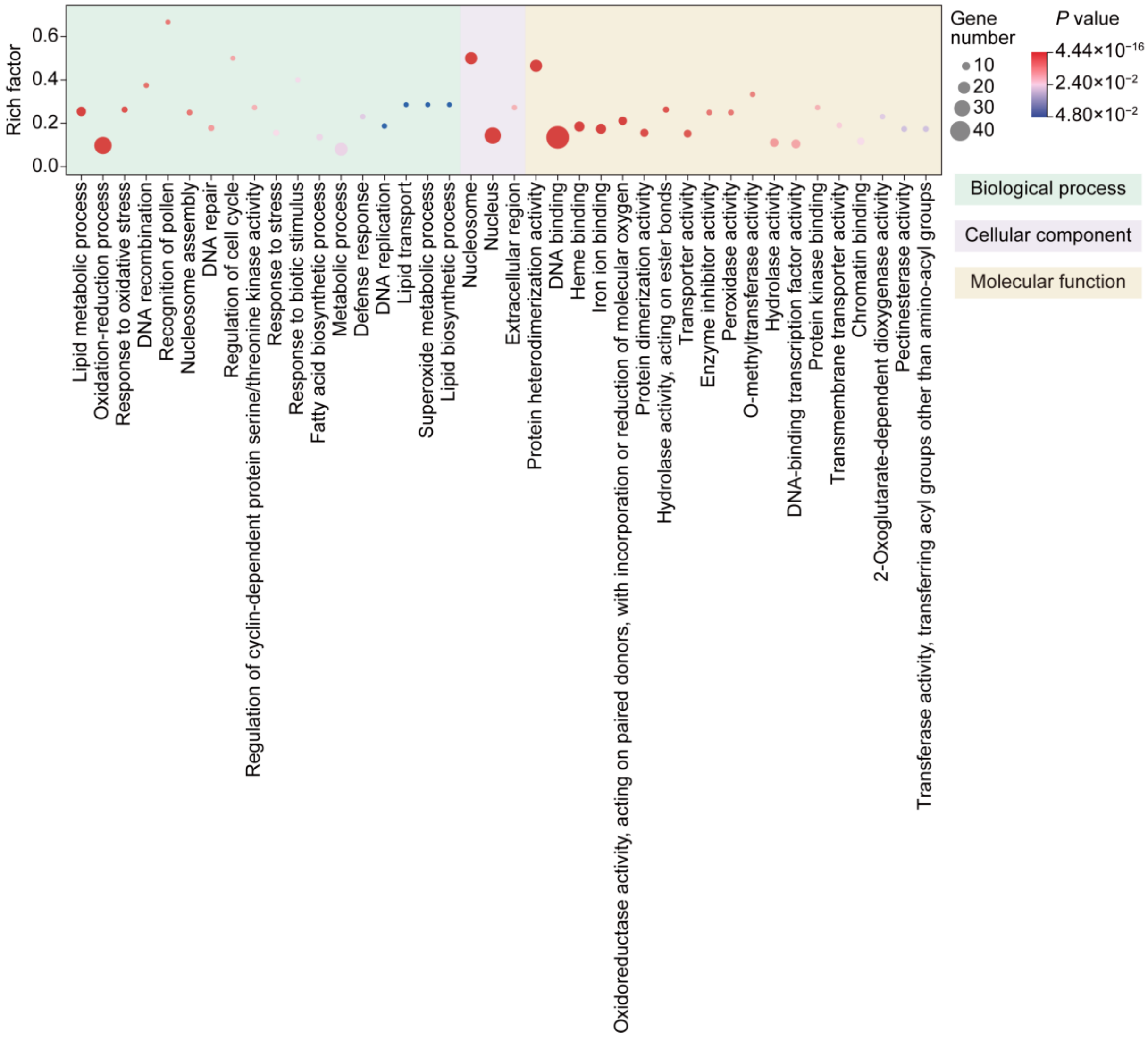

Figure S25. Enrichment analysis conducted on GO terms of DEGs in the liver tissues from PSMN+NIR group and LF group.

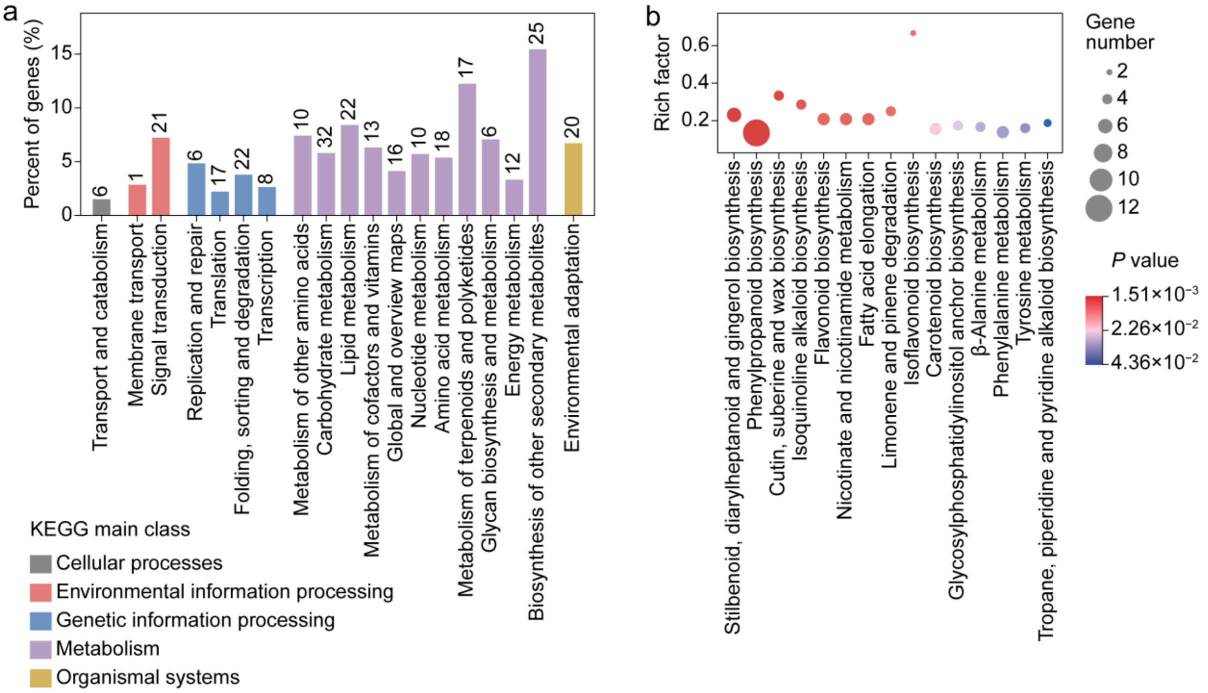

Figure S26. Enrichment analysis performed on KEGG main classes and pathways of DEGs in the liver tissues from PSMN+NIR group and LF group. a) Percent of DEGs enriched in various pathways assigned to different KEGG main classes. b) Rich factor of DEGs in different KEGG pathways.

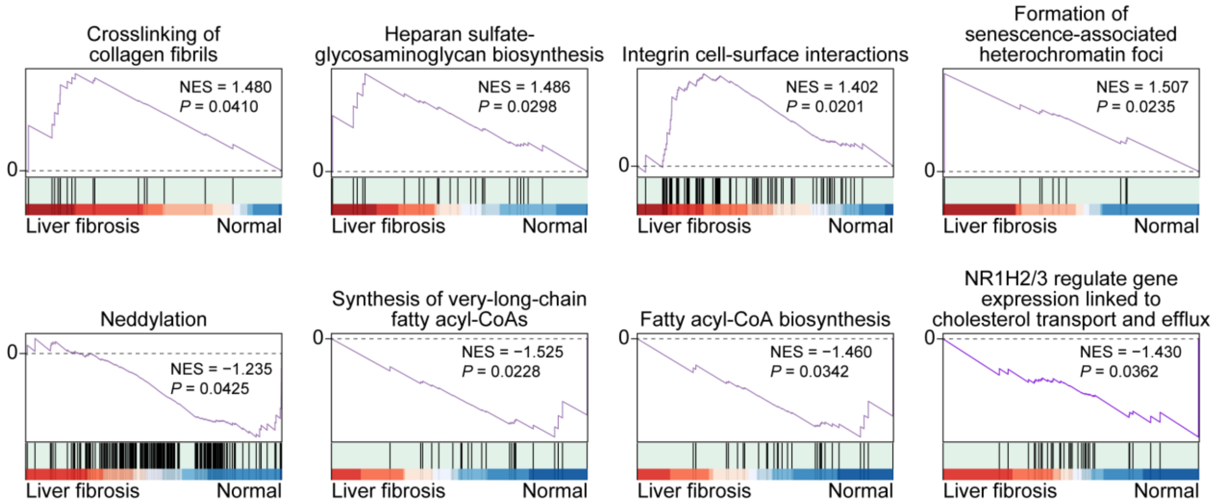

Figure S27. GSEA based on Reactome enrichments of DEGs in liver samples from LF group and normal group, including upregulated ($P < 0.05$ and NES > 1) and downregulated ($P < 0.05$ and NES < –1) pathways involved in ECM deposition and degradation, and HSC activation and quiescence.

Table S1 Primer sequences used in RT-qPCR assays.

Species	Name	Sequence (5'→3')
Mouse	*Casp3* F	ATGGAGAACAACAAAACCTCAGT
Mouse	*Casp3* R	TTGCTCCCATGTATGGTCTTTAC
Mouse	*Pcna* F	TTTGAGGCACGCCTGATCC
Mouse	*Pcna* R	GGAGACGTGAGACGAGTCCAT
Mouse	*Nos2* F	GTTCTCAGCCCAACAATACAAGA
Mouse	*Nos2* R	GTGGACGGGTCGATGTCAC
Mouse	*Tnf* F	CCCTCACACTCAGATCATCTTCT
Mouse	*Tnf* R	GCTACGACGTGGGCTACAG
Mouse	*Il1b* F	GCAACTGTTCCTGAACTCAACT
Mouse	*Il1b* R	ATCTTTTGGGGTCCGTCAACT
Mouse	*Il10* F	GCTCTTACTGACTGGCATGAG
Mouse	*Il10* R	CGCAGCTCTAGGAGCATGTG
Mouse	*Mrc1* F	CTCTGTTCAGCTATTGGACGC
Mouse	*Mcr1* R	CGGAATTTCTGGGATTCAGCTTC
Mouse	*Arg1* F	CTCCAAGCCAAAGTCCTTAGAG
Mouse	*Arg1* R	AGGAGCTGTCATTAGGGACATC
Mouse	*Tgfb1* F	CTCCCGTGGCTTCTAGTGC
Mouse	*Tgfb1* R	GCCTTAGTTTGGACAGGATCTG
Mouse	*Acta2* F	GTCCCAGACATCAGGGAGTAA
Mouse	*Acta2* R	TCGGATACTTCAGCGTCAGGA
Mouse	*Col1a1* F	GCTCCTCTTAGGGGCCACT
Mouse	*Col1a1* R	CCACGTCTCACCATTGGGG
Mouse	*Timp2* F	TCAGAGCCAAAGCAGTGAGC
Mouse	*Timp2* R	GCCGTGTAGATAAACTCGATGTC

Species	Name	Sequence (5'→3')
Mouse	*Gapdh* F	AGGTCGGTGTGAACGGATTTG
Mouse	*Gapdh* R	TGTAGACCATGTAGTTGAGGTCA
Human	*CASP3* F	CTTTTCATTATTCAGGCCTGCCG
Human	*CASP3* R	GGCACAAAGCGACTGGATGA
Human	*PCNA* F	ACTAAAATGCGCCGGCAATG
Human	*PCNA* R	AACTTTCTCCTGGTTTGGTGC
Human	*NOS2* F	GTGCAAACCTTCAAGGCAGC
Human	*NOS2* R	TGAGCCTCATGGTGAACACG
Human	*IL10* F	TCAAGGCGCATGTGAACTCC
Human	*IL10* R	GATGTCAAACTCACTCATGGCT
Human	*TGFB1* F	GGCCAGATCCTGTCCAAGC
Human	*TGFB1* R	GTGGGTTTCCACCATTAGCAC
Human	*MMP9* F	TGTACCGCTATGGTTACACTCG
Human	*MMP9* R	GGCAGGGACAGTTGCTTCT
Human	*ACTA1* F	AGCCCTCCTTCATCGGTATG
Human	*ACTA1* R	TCAGCGATCCCAGGGTACAT
Human	*COL1A2* F	CTGGTCTCGGTGGGAACTTT
Human	*COL1A2* R	AGCAGGTCCTTGGAAACCTT
Human	*FGF2* F	AGTGTGTGCTAACCGTTACCT
Human	*FGF2* R	ACTGCCCAGTTCGTTTCAGTG
Human	*PDGFB* F	CCTGTCTCTCTGCTGCTACC
Human	*PDGFB* R	CAATGGTCAGGGAACCCAGG
Human	*EDN1* F	GCCAAGGAGCTCCAGAAACA
Human	*EDN1* R	GAACAACGTGCTCGGGAGT

Species	Name	Sequence (5’→3’)
Human	*TIMP1* F	GCCTTCTGCAATTCCGACCT
Human	*TIMP1* R	TGGAACCCTTTATACATCTTGGTC
Human	*TIMP2* F	GCAACAGGCGTTTTGCAATG
Human	*TIMP2* R	TCTCAGGCCCTTTGAACATCTTT
Human	*GAPDH* F	GGAGCGAGATCCCTCCAAAAT
Human	*GAPDH* R	GGCTGTTGTCATACTTCTCATGG
Rat	*Acta2* F	CATCCGACCTTGCTAACGGA
Rat	*Acta2* R	AGTCCAGCACAATACCAGTTGT
Rat	*Pdgfa* F	ATAGACTCCGTAGGGGCTGA
Rat	*Pdgfa* R	GTCCTGGTCTTGCAAACTGC
Rat	*Pdgfb* F	CTACCTGCGTCTGGTCAGC
Rat	*Pdgfb* R	GCTCAGCCCCATCTTCGTCTAC
Rat	*Fgf2* F	CTTCTTCCTGCGCATCCATCC
Rat	*Fgf2* R	TCTTCTGTAACACACTTAGAAGCCA
Rat	*Mmp13* F	AGCAGCTCCAAAGGCTACA
Rat	*Mmp13* R	GTTGGGGTCTTCATCTCCTGG
Rat	*Timp1* F	GTAAAGCCTGTAGCTGTGCC
Rat	*Timp1* R	GCGTCGAATCCTTTGAGCATCTTA
Rat	*Timp2* F	ATGCAGACGTAGTGATCAGG
Rat	*Timp2* R	GAGGGGGCCGTGTAGATAAAT
Rat	*Tgfb1* F	CTGCTGACCCCCACTGATAC
Rat	*Tgfb1* R	CGTTTGGGACTGATCCCATTG
Rat	*Gapdh* F	GTCGGTGTGAACGGATTTGG
Rat	*Gapdh* R	TCCCGTTGATGACCAGCTTC

References

[1] a) Y. Jin, J. Zhang, Y. Xu, K. Yi, F. Li, H. Zhou, H. Wang, H. F. Chan, Y.-H. Lao, S. Lv, Y. Tao, M. Li, *Bioact. Mater.* **2023**, *28*, 112; b) Y. Hu, Y. Xu, R. L. Mintz, X. Luo, Y. Fang, Y.-H. Lao, H. F. Chan, K. Li, S. Lv, G. Chen, Y. Tao, Y. Luo, M. Li, *Biomaterials* **2023**, *293*, 121942.

[2] Y. Fang, X. Luo, Y. Xu, Z. Liu, R. L. Mintz, H. Yu, X. Yu, K. Li, E. Ju, H. Wang, Z. Tang, Y. Tao, M. Li, *Adv. Sci.* **2023**, *10*, 2300899.

[3] a) X. OuYang, X. Xu, Q. Qin, C. Dai, H. Wang, S. Liu, L. Hu, X. Xiong, H. Liu, D. Zhou, *Adv. Mater.*, https://doi.org/10.1002/adma.202304514; b) L. Chen, R. Zhao, J. Shen, N. Liu, Z. Zheng, Y. Miao, J. Zhu, L. Zhang, Y. Wang, H. Fang, J. Zhou, M. Li, Y. Yang, Z. Liu, Q. Chen, *Adv. Mater.* **2023**, *35*, 2306281; c) T. Liu, B. Xiao, F. Xiang, J. Tan, Z. Chen, X. Zhang, C. Wu, Z. Mao, G. Luo, X. Chen, J. Deng, *Nat. Commun.* **2020**, *11*, 2788; d) C. Zheng, Q. Zhong, K. Yi, H. Kong, F. Cao, C. Zhuo, Y. Xu, R. Shi, E. Ju, W. Song, Y. Tao, X. Chen, M. Li, *Sci. Adv.* **2023**, *9*, eadh2413.
